# Dysregulated expression of monoacylglycerol lipase is a marker for anti-diabetic drug metformin-targeted therapy to correct impaired neurogenesis and spatial memory in Alzheimer's disease

**DOI:** 10.7150/thno.44962

**Published:** 2020-05-15

**Authors:** Charvi Syal, Jayasankar Kosaraju, Laura Hamilton, Anne Aumont, Alphonse Chu, Sailendra Nath Sarma, Jacob Thomas, Matthew Seegobin, F Jeffrey Dilworth, Ling He, Fredric E Wondisford, Robert Zimmermann, Martin Parent, Karl Fernandes, Jing Wang

**Affiliations:** 1 Regenerative Medicine Program, Ottawa Hospital Research Institute, Ottawa, ON, K1H 8L6, Canada.; 2 Department of Cellular and Molecular Medicine, Faculty of Medicine, University of Ottawa, Ottawa, ON, K1H 8M5, Canada.; 3 Research Center of the University of Montreal Hospital (CRCHUM), Montreal, QC H2X 0A9, Canada.; 4 CNS Research Group (GRSNC), Montreal, QC H3T 1J4, Canada.; 5 Department of Neurosciences, Faculty of Medicine, Université de Montréal, Montreal, QC H3T 1J4, Canada.; 6 Departments of Pediatrics and Pharmacology, Johns Hopkins Medical School, Baltimore, MD, 21287, USA.; 7 Department of Medicine, Rutgers-Robert Wood Johnson Medical School, New Brunswick, NJ, 08901, USA.; 8 Institute of Molecular Biosciences, University of Graz, A-8010 Graz, Austria.; 9 CERVO Brain Research Centre, Department of Psychiatry and Neuroscience, Faculty of Medicine, Université Laval, Québec, QC, G1J 2G3, Canada.; 10 University of Ottawa Brain and Mind Research Institute, Ottawa, ON, K1H 8M5, Canada.; 11 Canadian Partnership for Stroke Recovery, Ottawa, ON, K1G 5Z3, Canada.

**Keywords:** monoacylglycerol lipase, metformin, adult neurogenesis, spatial memory, Alzheimer's disease

## Abstract

**Rationale:** Monoacylglycerol lipase (Mgll), a hydrolase that breaks down the endocannabinoid 2-arachidonoyl glycerol (2-AG) to produce arachidonic acid (ARA), is a potential target for neurodegenerative diseases, such as Alzheimer's disease (AD). Increasing evidence shows that impairment of adult neurogenesis by perturbed lipid metabolism predisposes patients to AD. However, it remains unknown what causes aberrant expression of Mgll in AD and how Mgll-regulated lipid metabolism impacts adult neurogenesis, thus predisposing to AD during aging. Here, we identify Mgll as an aging-induced factor that impairs adult neurogenesis and spatial memory in AD, and show that metformin, an FDA-approved anti-diabetic drug, can reduce the expression of Mgll to reverse impaired adult neurogenesis, prevent spatial memory decline and reduce β-amyloid accumulation.

**Methods:** Mgll expression was assessed in both human AD patient post-mortem hippocampal tissues and 3xTg-AD mouse model. In addition, we used both the 3xTg-AD animal model and the *Cbp*S436A genetic knock-in mouse model to identify that elevated Mgll expression is caused by the attenuation of the aPKC-CBP pathway, involving atypical protein kinase C (aPKC)-stimulated Ser436 phosphorylation of histone acetyltransferase CBP through biochemical methods. Furthermore, we performed *in vivo* adult neurogenesis assay with BrdU/EdU labelling and Morris water maze task in both animal models following pharmacological treatments to show the key role of Mgll in metformin-corrected neurogenesis and spatial memory deficits of AD through reactivating the aPKC-CBP pathway. Finally, we performed *in vitro* adult neurosphere assays using both animal models to study the role of the aPKC-CBP mediated Mgll repression in determining adult neural stem/progenitor cell (NPC) fate.

**Results:** Here, we demonstrate that aging-dependent induction of Mgll is observed in the 3xTg-AD model and human AD patient post-mortem hippocampal tissues. Importantly, we discover that elevated Mgll expression is caused by the attenuation of the aPKC-CBP pathway. The accumulation of Mgll in the 3xTg-AD mice reduces the genesis of newborn neurons and perturbs spatial memory. However, we find that metformin-stimulated aPKC-CBP pathway decreases Mgll expression to recover these deficits in 3xTg-AD. In addition, we reveal that elevated Mgll levels in cultured adult NPCs from both 3xTg-AD and *Cbp*S436A animal models are responsible for their NPC neuronal differentiation deficits.

**Conclusion:** Our findings set the stage for development of a clinical protocol where Mgll would serve as a biomarker in early stages of AD to identify potential metformin-responsive AD patients to restore their neurogenesis and spatial memory.

## Introduction

A key biological feature of the aging brain is its reduced ability to maintain and repair itself 1, which is associated with a decline in adult neurogenesis and memory 2-6. While the aging process in the context of neural stem cell function starts early in young adults 1,5,7, many factors, including aging-related epigenetic changes, are triggered to maintain homeostatic neurogenesis, which is responsible for new memory formation throughout adulthood 8. However, when these homeostatic mechanisms are perturbed, it may trigger a pathological aging process, predispose patients to Alzheimer's Disease (AD), and cause impaired neurogenesis and memory decline.

Monoacylglycerol lipase (Mgll) is a lipid hydrolase that breaks down the endocannabinoid 2-arachidonoyl glycerol (2-AG) to produce arachidonic acid (ARA) (and ARA-derived proinflammatory eicosanoids). Inhibition of Mgll activity not only enhances 2-AG levels, but also reduces ARA and ARA-derived proinflammatory eicosanoid levels 9. Since Mgll inhibitors provide many of the beneficial effects observed with direct cannabinoid receptor agonists or cyclooxygenase inhibitors without exerting their respective unwanted side-effects, several animal studies have reported Mgll as a promising therapeutic target for AD to ameliorate AD-associated neuropathology and memory decline 10,11. Interestingly, a recent report shows that 2-AG/eCBR signaling exhibits an age-dependent decline in activity that is associated with cognitive impairment 12,13. Despite the promising therapeutic potential of Mgll against AD, there are no FDA-approved drugs targeting Mgll other than a couple of Mgll inhibitors currently in Phase II clinical trials 14. Furthermore, there is little information as to the mechanisms governing Mgll expression, and how such pathways may be (mis)regulated in the context of aging or AD. Understanding the molecular control of lipid metabolism in regulating adult neurogenesis during normal aging and AD will thus provide fundamental knowledge for developing promising treatments for AD.

Adult neurogenesis is an intricate, multistep process that involves adult neural stem and progenitor cell (NPC) proliferation, differentiation, migration, and incorporation of the newborn neurons into functional neuronal circuitry. Each step of this complex process is tightly regulated by a variety of intrinsic factors and extrinsic cues including signaling cascades, epigenetic regulators and transcription factors 15. The hippocampus, a brain region vulnerable to neuronal damage at early stages of AD, is critical for learning and memory. The birth and integration of adult-born neurons that develop during the process of hippocampal neurogenesis play a key role in learning and memory 16-19, significantly contributing to spatial-navigation learning and long-term spatial memory retention, spatial pattern discrimination, contextual fear conditioning, clearance of hippocampal memory traces and reorganization of memory to extra-hippocampal substrates 20. Increasing studies show that hippocampal neurogenesis is affected in AD patients and animal models of AD 21-23. Interestingly, a recent paper shows that adult hippocampal neurogenesis is abundant in neurologically healthy subjects and drops sharply in patients with AD 23. This suggests that impaired neurogenesis is a potentially relevant mechanism underlying memory deficits in AD that might be amenable to novel therapeutic strategies. However, little research has focused on delineating the underlying molecular mechanisms contributing to the differences between normal physiological and AD-associated pathological aging in terms of hippocampal neuronal differentiation and maturation.

Since the aging process is characterized by widespread changes in the epigenetic landscape, it is plausible to hypothesize that dysregulated epigenetics during aging could contribute to AD predisposition. In this regard, our recent work identifies the role of atypical protein kinase C (aPKC) mediated Ser 436 phosphorylation in CBP, a histone acetyltransferase (HAT), as an important mechanism that sustains hippocampal neuronal differentiation, maturation, and memory during normal aging 8. We have also demonstrated that metformin, an FDA-approved anti-diabetic drug, can activate the aPKC-CBP pathway to promote NPC neuronal differentiation and improve hippocampus-associated memory *in vivo* 24,25. However, several clinical studies reveal controversial results on the effects of metformin on cognitive decline and AD 26-29, which might be due to the heterogeneity of pathological aging associated with AD 30. Therefore, identifying the exact molecular targets through which metformin acts to promote cognition is vital. These molecular targets have the potential to be used as a theranostic marker to screen a subpopulation of AD patients for effective and early treatment with metformin to bring personalised medicine to clinical practice.

In the present study, we show that aging-dependent induction of Mgll is observed in the 3xTg-AD mouse model and in human AD patient post-mortem hippocampal tissues. In addition, we reveal that 3xTg-AD mice exhibit an impaired aPKC-CBP pathway that leads to increased Mgll expression, associated with perturbed adult neuronal differentiation and spatial memory deficits. Interestingly, activation of the aPKC-CBP pathway by metformin, an FDA-approved drug, directly represses Mgll expression to promote NPC differentiation at the expense of proliferation. Importantly, metformin treatment *in vivo* in 3xTg-AD mice corrects the impaired aPKC-*Cbp* pathway to repress Mgll expression, significantly rescuing impaired adult neurogenesis, preventing spatial memory decline and reducing β-amyloid accumulation.

## Materials and Methods

### Study design

The research objective of this study was to test the role of Mgll in regulating aging-dependent AD pathophysiology using an animal model of AD and to examine how metformin-stimulated epigenetic pathway represses Mgll expression to rescue neurogenesis and spatial memory using transgenic and AD mouse models. We tested the role of Mgll in regulating aging-dependent AD pathophysiology including adult neurogenesis and spatial memory using 3xTg-AD mice and examined the effect of metformin on the reduction of Mgll expression to rescue adult neurogenesis and spatial memory deficits in 3xTg-AD mice. We chose three main endpoints: quantification of *in vivo* adult hippocampal neuronal differentiation, measurement of spatial memory, and changes in aPKC signaling and Mgll expression throughout aging development and primary NPC cultures from genotyped animals. Randomization and blinding of experimenters were done throughout the study. Experiments were carried out in at least three biological replicates. The work reported here followed the ARRIVE guidelines for animal studies. Animal experiments were performed in accordance with the guidelines of the Canadian Council on Animal Care and stipulations of the Ethics Board and the Animal Care Committee at the University of Ottawa.

### Animals

All animal use was approved by the Animal Care Committees of the University of Ottawa in accordance with the Canadian Council of Animal Care policies. Transgenic mouse lines, *Cbp*S436A, Non-Tg, 3xTg-AD and Mgll-flxed, were maintained on a 12 h light/12 h dark cycle with *ad libitum* access to food and water. Only wild type (WT) and homozygous (*Cbp*S436A) mice 31 were used for experiments and mice heterozygous for *Cbp*S436A were used for breeding. 3xTg-AD (B6;129-Psen1tm1MpmTg (APPSwe, tauP301L)1Lfa/Mmjax) and non-transgenic, Non-Tg (B6129SF2/J) mice were purchased from the Jackson Laboratory. Both male and female mice were used in the experiments. Each set of experiments was performed with littermates or the same age of mice. The generation of the transgenic *Cbp*S436A, 3xTg-AD and Mgll-flxed lines has been described previously 31-33.

### NIH3T3 cell culture

NIH3T3 cells, gifted by Dr. Xiaohui Zha (Ottawa Hospital Research Institute, Ottawa, Canada), were cultured in high glucose Dulbecco's Modified Eagle's Medium (DMEM) (Wisent Bioproducts, 319-005-CL) containing 10% fetal bovine serum (FBS) (Life Technologies, 12484010) and 1% penicillin-streptomycin (Thermo Fisher, 15140122). The cells were maintained in a humidified incubator at 37 °C with 5% CO_2_ and passaged every three days. For transfection, the cells were seeded in 6-well culture plates at 500,000 cells per well. Following overnight incubation, cells were transfected with pSUPER.retro.neo-*Mgll* shRNA 1 (Forward:5'GAT CCC CCG TTA TGA TGA GCT GGC TCT TCA AGA GAG AGC CAG CTC ATC ATA ACG TTT TTA-3'; Reverse: 5'AGC TTA AAA ACG TTA TGA TGA GCT GGC TCT CTC TTG AAG AGC CAG CTC ATC ATA ACG GGG-3') (2.5 μg), pSUPER.retro.neo-*Mgll* shRNA 2 (Forward: 5'GAT CCC CGG CTG GAC ATG CTG GTA TTT TCA AGA GAA ATA CCA GCA TGT CCA GCC TTT TTA-3'; Reverse: 5'AGC TTA AAA AGG CTG GAC ATG CTG GTA TTT CTC TTG AAA ATA CCA GCA TGT CCA GCC GGG-3') (2.5 μg), pSUPER.retro.neo-*Mgll* shRNAs 1 and 2 (1.25 μg for each), or a non-specific scrambled (Scr) pSUPER.retro.neo-Scr shRNA (Forward: 5'GAT CCC CCT TCC TCT CTT TCT CTC CCT TGT GAT TCA AGA GAT CAC AAG GGA GAG AAA GAG AGG AAG TTT TTA-3'; Reverse: 5'AGC TTA AAA ACT TCC TCT CTT TCT CTC CCT TGT GAT CTC TTG AAT CAC AAG GGA GAG AAA GAG AGG AAG GGG-3') (2.5 μg), mixed with 7.5 μL of TransIT-X2® Dynamic Delivery System (Mirius, MIR6003) in Opti-MEM™ (Thermo Fisher, 31985062) per well. Cells were harvested 48 h later, and the knockdown efficiency was assessed using a western blot.

### SVZ neurosphere culture and GPR40 antagonist treatment

Subventricular zone (SVZ) tissues were microdissected from the subependyma of lateral ventricles of 6 to 8 weeks old mice (WT, *Cbp*S436A, Non-Tg, 3xTg-AD and Mgll-flxed) according to the previous publication 24. The collected tissues were digested in 5-10 mL enzyme mix containing 40 mg trypsin (Sigma-Aldrich, T1005), 25 mg hyaluronidase (Sigma-Aldrich, H3884-500) and 3-5 mg kynurenic acid (Sigma-Aldrich, K3375) suspended in 30 mL HBSS, for 40 min at 37 °C while mixing on a HulaMixer™ Sample Mixer. Digestion was stopped using a sterilized trypsin inhibitor (0.67 mg/mL) (Sigma-Aldrich, T2011-500) prepared in serum-free medium (SFM). The tissues were mechanically dissociated into single cell suspensions by passing through P1000 and P200 pipettes and cells were collected by centrifuging twice at 1500 rpm for 5 min. The cell pellet was resuspended in supplemented SFM containing 20 ng/mL epidermal growth factor (EGF) (Sigma-Aldrich, E9644), 20 ng/mL basic fibroblast growth factor (bFGF) (PeproTech, 100-18B), 2 μg/mL heparin (Sigma-Aldrich, H3149-100KU) and 1x B-27™ supplement (B) (Thermo Fisher Scientific, 17504-044). Live cells were counted using trypan blue (Thermo Fisher Scientific, 15250061), plated at 10 cells/μL in 6-well plates, and cultured for seven days *in vitro* (DIV) without disturbance in a humidified incubator at 37 °C with 5% CO_2_ to allow neurosphere (NS) formation. Free-floating primary NS were collected and centrifuged at 1500 rpm for 5 min. The cell pellets were resuspended and dissociated into single-cell suspensions by triturating in supplemented SFM. The cells were filtered through a cell strainer (40 μm pore size), counted, and seeded at 2 cells/μL in 6 well plates to initiate secondary NS formation. Six days later, the secondary NS or second passage (P2) NPCs were collected and passaged until the fifth passage (P5) or used for further experiments as described below.

DC 260126 (Tocris, 5357-10), a GPR40 antagonist was prepared in dimethyl sulfoxide (DMSO) (Sigma-Aldrich, D2650) as a 1 mM stock and stored at -20 °C. P2 3xTg-AD and Non-Tg, and P5 WT and *Cbp*S436A NPCs were treated with 100 nM DC 260126 or 0.01% DMSO (control) at time of plating (2 cells/ul in 6-well plates), and neurospheres were imaged and assessed six days later as described below.

### SVZ NPC transfection, conditioned medium treatment and differentiation

P2 or P5 WT, *Cbp*S436A, Non-Tg, 3xTg-AD and *Mgll*-flxed NPCs were plated on PLO- laminin coated coverslips at 1x10^5^cells per well in 24-well plates, in supplemented SFM. Following overnight incubation, each well was transfected with either 0.4 μg *Mgll* shRNA 1 and 2 (0.2 μg for each) or 0.4 μg phosphomimic *Cbp*S436D plasmid 34 together with 0.2 μg of a CAG-eGFP (eGFP) plasmid, or 0.6 μg CAG-Cre:GFP plasmid 35 (Addgene plasmid # 13776), mixed with 1.8 μL of TransIT-X2® Dynamic Delivery System (Mirius, MIR6003) in Opti-MEM™ (Thermo Fisher, 31985062). In these experiments, non-specific Scramble shRNA, pcDNA3.1 empty vector 34 and eGFP plasmid were transfected in separate wells as controls for *Mgll* shRNA, *Cbp*S436D plasmid and CAG-Cre:GFP plasmid, respectively. 24 h following transfection, the medium was switched to differentiation medium (SFM + 10% FBS), with or without 1 μM metformin. Conditioned medium (CM) from differentiating WT and *Cbp*S436A NPCs was collected seven days upon differentiation, centrifuged at 13,000 rpm for 10 min, and stored at -20 °C for later use, while transfected cells were assessed using immunocytochemistry as described below.

Another set of P2 WT and *Cbp*S436A NPCs were plated on PLO-laminin coated coverslips at 1x10^5^cells per well in 24-well plates in supplemented SFM. Following overnight incubation, the medium was changed to differentiation medium containing 50% of the previously collected CM and supplemented with 1 μM metformin. The NPCs were assessed seven days later using immunocytochemistry as described below.

### Drug treatment in cultured SVZ NPCs

Mgll inhibitor, JZL 184 (Cayman Chemical, 13158-5) and eCBR1 and eCBR2 agonists, Arachidonyl-2'-chloroethylamide (ACEA) (Cayman Chemical, 91054) and JWH-133 (Tocris, 1343-10) respectively, and eCBR1 and eCBR2 antagonists, AM251 (Cayman Chemical, 71670) and AM630 (Cayman Chemical, 10006974) were prepared in DMSO as a 1 mM stock, while metformin (Sigma- Aldrich, D150959-5G) was prepared as a 0.5 mM stock in sterilized water. All reagents were stored at -20 °C until required.

P2 3xTg-AD and Non-Tg NPCs were plated on PLO-laminin coated coverslips in a 24-well plate at 1x10^5^cells per well and cultured in SFM containing 10% FBS. The medium was supplemented with 500 nM metformin, 1 μM JZL 184, 1 μM ACEA or 1 μM JWH-133. Water treatment or 0.1% DMSO were used as control groups. The differentiating NPCs were cultured for seven days and assessed by immunocytochemistry as described below. P5 WT and *Cbp*S436A NPCs transfected with *Mgll*-shRNAs and eGFP plasmid or Scr-shRNA and eGFP plasmid, and P2 *Mgll*-flxed NPCs transfected with either Cre:GFP (Addgene #13776) or eGFP plasmids, were cultured in SFM containing 10% FBS. The medium was supplemented with 1 μM eCBR1 antagonist (AM251), 1 μM eCBR2 antagonist (AM630) or 0.1% DMSO (control groups). The differentiating NPCs were cultured for seven days and assessed by immunocytochemistry as described below.

### RNA sequencing analysis

Total RNA was isolated from P2 differentiating WT and *Cbp*S436A NPCs cultured in the presence of metformin (1 μM) for 6 days (1x10^6^ cells/genotype, n=2 per genotype) using a RNeasy Mini Kit (Qiagen, 74104) including a step of on-column DNAse I digestion. Single-end sequencing was performed at the McGill University and Génome Québec Innovation Centre (Montréal, QC) on an Illumina HiSeq 2000.

RNA sequencing data was analyzed using a pipeline consisting of the Bowtie, TopHat, CuffLinks and CummeRbund software suites 36. In this pipeline, Bowtie version 2.0.2 37 was used to map reads to the mm9 genome (UCSC) and known mm9 transcripts (RefSeq mm9 build37.2) using default parameters. TopHat version 2.0.6 38 allowed for the identification of splice junctions using default values with the exception of r = 0. Transcript assembly and quantification was performed with CuffLinks version 2.0.2 39 using default values. CummeRbund bioconductor package (http://compbio.mit.edu/cummeRbund/) was then used to identify differential gene expression. Genes with a q-value ≤ 0.05 were considered to be significant. RNA sequencing data has been deposited in GEO. The accession number is GSE127730.

### RNA extraction, cDNA synthesis, and quantitative real-time polymerase chain reaction

Differentiating WT and *Cbp*S436A NPCs cultured in the presence of metformin (1 μM) were scraped from culture plates and snap frozen in liquid nitrogen. RNA was extracted from these NPCs using TRIzol plus PureLink™ RNA Mini Kit (Invitrogen, 12183018A). cDNA was synthesized from RNA using the QuantiTect Reverse Transcription Kit from Qiagen. Quantitative real-time polymerase chain reaction (qPCR) was carried out using the Sensifast^tm^ SYBR-green master mix (Bioline) and 400 nM primers (final concentration) on the Stratagene MX3000 using MXPro qPCR software. Cycling parameters were: 95 °C for 2 min followed by 40 cycles of 95 °C (15 s), 58 °C (10 s), and 72 °C (20 s) ending with a melting curve analysis to assess the amplification of a single amplicon. All reactions were performed in duplicate, with the median cycle time used for analysis. GAPDH was used as a housekeeping gene against all experimental genes. Primer sequences as following:* Mgll* forward 5'- CGG ACT TCC AAG TTT TTG TCA GA -3', reverse 5'- GCA GCC ACT AGG ATG GAG ATG - 3'*. Gapdh* forward 5'-AGG TCG GTG TGA ACG GAT TTG - 3', reverse 5'- TGT AGA CCA TGT AGT TGA GGT CA - 3'.

### Chromatin immunoprecipitation (ChIP)

*Cbp* binding to *Mgll* promoters in SVZ NPCs was measured using a crosslink ChIP protocol as previously described 40. Briefly, 1x10^6^ differentiating WT and *Cbp*S436A NPCs cultured in the presence of metformin were collected and cross-linked with 1% (w/w) final formaldehyde for 30 min at room temperature followed by sonication using a Bioruptor (Diagenode). CBP antibody (Santa- Cruz, sc-583) was coupled to Dynabeads with protein A (for rabbit antibodies) for 2 h at room temperature in IP buffer containing 100 mM KCl. Resin-bound antibodies were then washed extensively with sonication buffer (50 mM HEPES, pH 7.9, 140 mM NaCl, 1 mM EDTA, 1% (w/v) Triton X-100, 0.1% (v/v) sodium deoxycholate, 0.1% (w/v) SDS and protease inhibitors) and equilibrated with 1 mL sonication buffer containing 2 µg sonicated λ DNA and 1 mg/mL ovalbumin, before incubation with the precleared chromatin overnight at 4 °C. ChIP real- time qPCR analysis was done on a Stratagene MX3000 using MXPro qPCR software using primers for Mgll: forward 5'- CCT GCC TCA GGA TAG GAG CC - 3', reverse 5'- CGA GCA AAG TCA CCC CGA TTC T -3', and primers for IgHy enhancer (inactive loci): forward 5'- ACC CTG GGA AGA CCA TAC TTA ATCT -3', reverse 5'-CCA TCC ACA CTC GTG CCT TA-3'.

### Immunocytochemistry

Cells growing on PLO-laminin coated glass coverslips were fixed with 4% paraformaldehyde (PFA) (Sigma-Aldrich, 158127) for 10 min, blocked, and permeabilized with 10% normal goat serum (NGS, Invitrogen, 10000C) and 0.3% TritonX-100 (Fisher Scientific BP151-100) in 1x PBS buffer. Fixed cells were then incubated with primary antibodies at 4 ºC overnight, with secondary antibodies at room temperature for 1 h, counterstained with Hoechst 33342 (1:1000, Cell Signalling Technology, 4082S) and mounted with Permafluor (Thermo Fisher TA-030-FM).

The primary antibodies used were: chicken anti-GFP (1:2000, Abcam, ab13970), rabbit anti-AMPK (1:250, Abcam, ab32047), mouse anti-Sox2 (1:500, Abcam, ab79351) and mouse anti-βIII tubulin (1:500, Biolegend, 801201). Alexa Fluor-conjugated secondary antibodies used were: goat anti-chicken Alexa Fluor 488 (1:500, Thermo Fisher Scientific, A11039), donkey anti-rabbit Alexa Fluor 488 (1:500, Thermo Fisher Scientific,21206) and donkey anti-mouse Alexa Fluor 555 (1:500, Thermo Fisher Scientific, A31572), diluted in 0.3% Triton X-100 solution in 1x PBS.

### JZL 184, metformin and 5-Bromo-2'-deoxyuridine (BrdU)/ 5-Ethynyl-2'-deoxyuridine (EdU) injections

JZL 184 (Cayman Chemical, 13158-50) was prepared in DMSO at 40 mg/mL and stored at -20 °C. Dissolved JZL 184 was mixed with 2.5% Tween-80 in 1x PBS and administered the same day. 6-month-old WT and *Cbp*S436A mice received daily intraperitoneal (i.p., 8 mg/kg) injections of JZL 184 or saline containing 2.5% Tween-80 and 2% DMSO as a vehicle control for 14 days. To label dividing cells, the mice also received two i.p. injections of BrdU (Sigma- Aldrich, B9285-1G, 100 mg/kg), 4 h apart, for three consecutive days, after an initial two days of treatment with JZL 184 alone.

Metformin (Sigma-Aldrich, D150959) was prepared in PBS at 20 mg/mL and stored at -20 °C. 8 to10 month-old 3xTg-AD and Non-Tg mice received daily i.p. injections (200 mg/kg) of metformin or PBS (as a control) for 14 days. To label dividing cells, the mice also received two i.p. injections of EdU (ChemCruz, SC-284628, 50 mg/kg), 4 h apart, for three consecutive days, after an initial two days of treatment with metformin alone.

### Tissue preparation and immunohistochemistry

Following 14 days of drug treatment, mice were anesthetized with 0.1 mL sodium pentobarbital (65 mg/mL, i.p.), and transcardially perfused with 4% PFA in 1x PBS. The brains were extracted and post-fixed in 4% PFA overnight at 4 °C, followed by storage in 30% sucrose solution containing 0.01% sodium azide (Fisher Scientific, 19038) for at least 48 h. Samples were covered in optimal cutting temperature solution (VWR, 95057-838) and flash frozen on dry ice. Serial 20 μm hippocampal sections were obtained using a cryostat (Leica Biosystems, CM1850) and sequentially mounted on glass slides, to encompass the entire hippocampus. Sections were stored at -80°C until required.

Brain sections were fixed with 4% PFA for 15 min. For WT and *Cbp*S436A, the brain sections were treated with 1 N HCl at 45 °C for 1 h and then extensively washed with 1x PBS. Subsequently, the sections were blocked/permeabilized for at least 1 h at room temperature in 10% normal horse serum (NHS) (Thermo Fisher Scientific, 16050122) with 0.3% Triton X-100 prepared in 1x PBS. The brain sections were sequentially incubated with the primary antibody prepared in 10% NHS with 0.3% Triton X-100 in 1x PBS, at 4 °C overnight. The antibodies used were: goat anti-DCX (1:200, Santa-Cruz SC-8066), rat anti-BrdU (1:200, AbD Serotec, OBTOO30G) and mouse anti-NeuN (1:500, Millipore, MAB377). Following overnight incubation for each primary antibody, the sections were washed three times for 5 min each with 1x PBS, before addition of an Alexa Fluor-conjugated secondary antibody: donkey anti-goat Alexa Fluor 555 (1:500, Thermo Fisher Scientific, A-21432) donkey anti-rat Alexa Fluor 488 (1:500, Thermo Fisher Scientific, A21208) or goat anti-mouse Alexa Fluor 647 (1:500, Thermo Fisher Scientific, A-21235), diluted in 0.3% Triton X-100 solution in 1x PBS for 1 h. The sections were then mounted using PermaFluor™ solution (Thermo Fisher Scientific, TA-030-FM).

For 3xTg-AD and Non-Tg, the brain sections were treated with a clearing solution containing 200 mM boric acid, 4% sodium dodecyl sulfate (pH 8.5) for 4-5 h. The sections were then washed twice with 0.3% Triton X-100 prepared in 1x PBS. Subsequently, the sections were blocked/permeabilized for at least 1 h at room temperature in 3% bovine serum albumin (BSA, Cell Signalling Technology, 9998S) in 0.3% Triton X-100 (Fisher Scientific BP151-100) prepared in 1x PBS. The brain sections were sequentially incubated with the primary antibody prepared in the blocking solution, at 4 °C overnight. The antibodies used were: goat anti-DCX (1:200, Santa-Cruz SC-8066) and mouse anti-NeuN (1:500, Millipore, MAB377). Following overnight incubation, the sections were washed three times for 5 min each with 1x PBS, before addition of an Alexa Fluor-conjugated secondary antibody: donkey anti-goat Alexa Fluor 555 (1:500, Thermo Fisher Scientific, A-21432) or goat anti-mouse Alexa Fluor 647 (1:500, Thermo Fisher Scientific, A-21235), diluted in 0.3 % Triton X-100 solution in 1x PBS for 1 h. EdU staining was performed using Click-iT EdU kit (Thermo Fisher Scientific, C10340) according to the manufacturer's protocol. The sections were then mounted using PermaFluor™ solution (Thermo Fisher Scientific, TA-030-FM).

For brightfield immunohistochemistry, 3xTg-AD brain sections and human AD patient and age-matched healthy control DG sections (obtained from a human brain bank of the CERVO Brain Research Centre located in Quebec City, which required informed consent before donation) were treated with clearing solution containing 200 mN boric acid and 4% sodium dodecyl sulphate, pH 8.5 for 4-5 h. The sections were then washed twice with 0.3% Triton X-100 prepared in 1x PBS. Subsequently, the sections were blocked and permeabilized for at least 1 h at room temperature in 3% BSA in 0.3% Triton X-100 prepared in 1x PBS. The brain sections were then incubated with a rabbit anti-Mgll primary antibody (1:100, Abcam, ab24701) prepared in the blocking solution, at 4 °C overnight. The next day, the brain sections were washed three times for 5 min each with 1x PBS before the addition of an anti-rabbit biotinylated secondary antibody (1:500), prepared in 1:1 blocking solution mixed with 0.3% Triton X-100 in 1x PBS solution, at room temperature for 1 h. The sections were then washed twice for 5 min each with 1x PBS and incubated with an avidin-biotin complex solution (VECTASTAIN Elite ABC Kit, Vector Laboratories, PK-6100), diluted in 1:1 blocking solution mixed with 0.3% Triton X-100 in 1x PBS solution, for 1 h at room temperature. The sections were washed twice with 1x PBS and treated with a 3,3-diaminobenzidine (DAB)-containing solution for 2 min, followed by washing with 1x PBS for 10 min. The sections were then mounted on glass slides and once dry, dehydrated using sequentially increasing concentrations of ethanol (35%, 50%, 70%, 80%, 90%, 95% and 100%), and treated with CitriSolv (Fisher) before mounting using PermaFluor™ solution.

### Imaging and quantification

P2 and P5 neurospheres were imaged at 5x magnification, with Zeiss Axiovert 200M inverted microscope using Zeiss Axiovision software. Eight to ten images were randomly taken per treatment group and neurosphere diameters were measured using FIJI software. On average, 300-400 neurospheres were assessed per condition.

Fluorescent images were taken on a Zeiss Imager M.1 fluorescent microscope with Z-axis capability, using Zeiss Axiovision software. For *in vitro* transfection experiments, all GFP positive cells were exhaustively quantified (at least over 100 cells/per condition). For all other *in vitro* experiments six to eight images were taken randomly at 20 x magnification, and on average 600-800 cells were quantified per condition. Cell counts were done blinded to experimental groups, using FIJI software.

For *in vivo* hippocampal neurogenesis, digital image acquisition was performed using a Zeiss Imager M.1 fluorescent microscopy with Zeiss Axiovision software that contains z-axis capability. 10-15 images were captured in the Z-axis per section at a maximum of 1 µm apart and processed as an optical stack of 10-15 scanned slices for quantification. Every tenth section throughout the septotemporal axis of the hippocampus (-1.3 mm to -3.70 mm relative to bregma referring to the rostral-caudal coordinates) was quantified. Every BrdU and EdU positive cell within the dentate gyrus region including the SGZ, granular cell layer and hilus was exhaustively quantified using a modified stereological method that have been described before 8.

For brightfield microscopy, digital images were taken using a Zeiss Imager M2 microscope with Zeiss Zen software.

### Morris Water Maze (MWM) task

6-month-old WT and *Cbp*S436A (n = 10-11/ group) mice received JZL 184 or vehicle injections (i.p. 8 mg/kg) every 2-3 days for 14 days before starting MWM task as described below. In addition, JZL 184 or vehicle injections continued throughout the period of MWM task (Figure 3A). 7 to 8-month-old 3xTg-AD and Non-Tg (n = 13-23/group) mice received metformin or control (water) treatment by drinking water (4 mg/mL) for 6 weeks before starting MWM task as described below. Metformin treatments also continued throughout the period of MWM task (Figure 3F).

The MWM task was conducted at the University of Ottawa Behavioural Core. The mice were trained on the hidden platform version of the water maze using a circular pool (122 cm diameter, 83.5 cm depth, 22 ºC) filled with 74.2 cm of water and made opaque with a nontoxic white paint. The escape platform (10 cm diameter) was submerged 0.5 cm below the water surface. All testing was conducted under 120 lux lighting and an extra maze visual cue with “X” printed in black ink (2.9 cm thickness) on a white paper (13.5 x 15 cm) was located on one wall. Acquisition was measured as latency to reach the platform and four possible start locations were pseudo-randomly assigned to each trial. Each animal was given four 60 s trials to find the platform with a 20 min inter-trial interval across 4 days. A probe trial was then completed at day 5 and day 11 after the 4-day training, leaving the mice swim in the pool for 60 s when the platform was removed. Ethovision tracking software was used to record the behaviour of mice during testing.

### Blood cells collection

Non-Tg and 3xTg-AD ice were euthanized via i.p. injection of 0.15 mL of 65 mg/mL sodium pentobarbital. Immediately following full anesthetization, a lateral incision underneath the diaphragm and along the rib cage was performed to expose the thoracic cavity. Using a 27 G syringe, 0.3 mL blood was collected from the right atrium of the heart and subsequently mixed with 0.2 mg heparin to prevent coagulation. Samples were then immediately centrifuged at 300 g for 10 min at 4 °C. Following centrifugation, supernatant containing blood plasma was discarded, and samples containing blood cell pellet were then flash frozen in liquid nitrogen and stored at -80 °C until RNA extraction and isolation was performed as described previously.

### Western blot analysis and densitometry

P2 WT, *Cbp*S436A, Non-Tg and 3xTg-AD NPCs and P5 WT and *Cbp*S436A NPCs obtained from neurospheres were pelleted and snap frozen in liquid nitrogen for further western blot analysis. In addition, P2 WT and *Cbp*S436A neurosphere-derived NPCs were plated on PLO- laminin coated 6-well plates at 1x10^6^cells per well in differentiation medium: SFM containing 10% FBS, supplemented with 1 μM metformin. The differentiating NPCs were harvested seven days upon differentiation for western blot analysis. The hippocampal tissues were isolated from WT and *Cbp*S436A and Non-Tg and 3xTg-AD brains and snap frozen in liquid nitrogen for further western blot analysis.

Cultured cells or frozen hippocampal tissues were lysed in cold lysis buffer (25 mM Tris, pH = 7.4, 10 mM NaCl, 2 mM EDTA, 1 mM EGTA 0.5% Triton X-100, 10% glycerol) containing 1 mM sodium orthovanadate (Sigma-Aldrich S6508-10G), 20 mM sodium fluoride (Fisher Scientific, AC201295000), 1 mM PMSF (Sigma-Aldrich, P7626-5G), 10 μg/mL aprotinin (Fisher Scientific, PI78432) and 10 μg/mL leupeptin (Fisher Scientific, PI78436). The lysates were homogenized by trituration, and sonicated (three 5 s pulses with 1 min intervals), followed by centrifugation at 13000 rpm for 15 min at 4 °C. 100 μg protein lysates from hippocampal tissues and 20-30 μg protein lysates from cultured cells were resolved on a 12% SDS-PAGE gel, and western blot analysis was performed as previously described 8. Densitometry was performed using FIJI software.

Primary antibodies used in western blot analysis: rabbit anti-Mgll (1:1000, Abcam, ab24701), rabbit anti- p-aPKCζ/ι (phosphorylation at T410/403) (1:500, Cell Signaling Technology, 9378S), mouse anti-aPKCζ/ι (1:1000, BD Biosciences, 610175), mouse anti-CBP (1:100, Santa-Cruz Biotechnology, Sc-7300) and mouse anti-GAPDH (1:50,000, Abcam, ab8245) antibodies. The HRP-conjugated secondary antibody used in western blot analysis: goat anti-rabbit or anti-mouse (1:3000, Cell Signaling Technologies, 7074 and 7076, respectively).

### Immunoprecipitation

Hippocampal tissues from 3xTg-AD and Non-Tg were homogenized and lysed in lysis buffer (25 mM Tris, pH = 7.4, 10 mM NaCl, 2 mM EDTA, 1 mM EGTA, 0.5% Triton X-100, 10% glycerol) containing 1 mM PMSF, 1 mM sodium orthovanadate, 20 mM sodium fluoride, 10 µg/mL aprotinin and 10 µg/mL leupeptin. The extractions were sonicated 3 times with 5 s pulses at 1 min intervals. Then, 500 µg protein lysate from each sample was incubated with 50 μl protein A conjugated magnetic beads and 3 μg anti-CBP antibody (Santa-Cruz Biotechnology, Sc-583) or normal rabbit IgG antibody at 4 ºC overnight. Following that, the magnetic beads were rinsed 3 times with lysis buffer, boiled with sample buffer, and loaded on a 6-12% gradient SDS-PAGE gel.

### Statistical analysis

All data analysis was conducted using GraphPad Prism 6 software. Statistical analyses were performed using either a two-tailed Student's t-test, or one-way ANOVA with Dunnett's post-hoc test or two-ANOVA with Tukey's post-hoc test to analyze multiple groups, where appropriate. All values are expressed as mean ± standard error of the mean (SEM), unless stated otherwise. For all experiments, differences with P-value < 0.05 were considered statistically significant.

## Results

### Mgll is highly expressed in aging AD brain, associated with reduced aPKC activity

**Figure 1:**
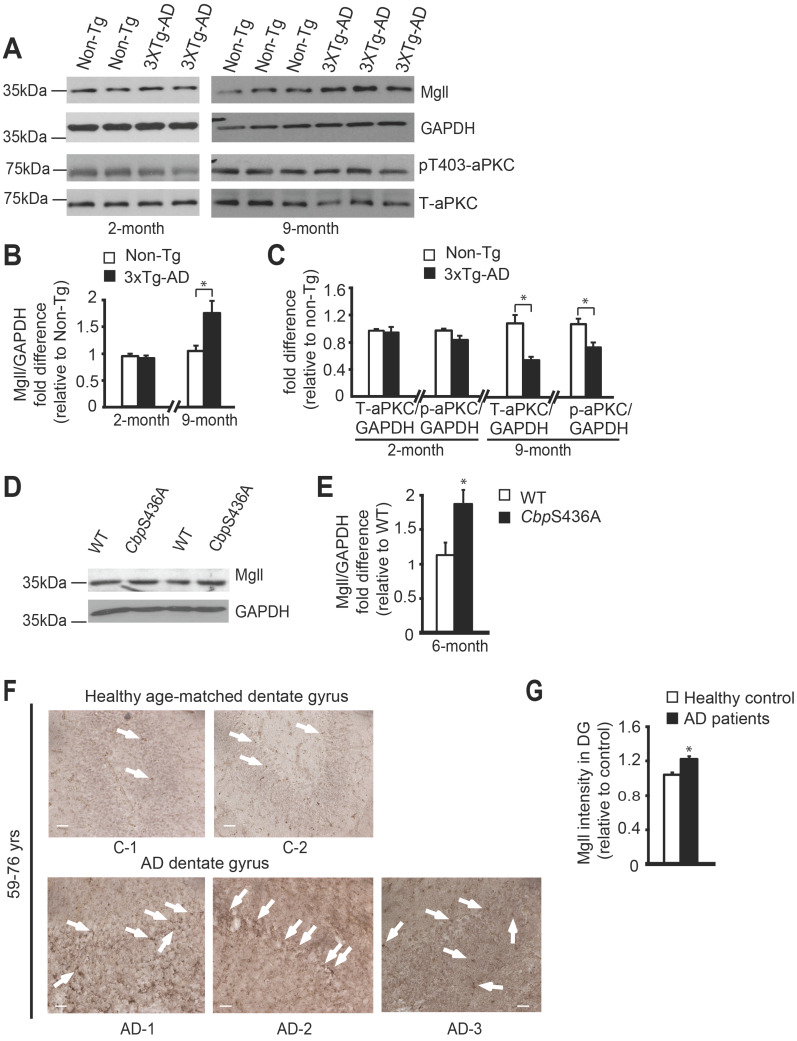
**Mgll is highly expressed in aging AD brain, associated with reduced aPKC activity. (A-C)** Representative western blot images and quantitative analysis of total protein lysates from 2-month and 9-month Non-Tg and 3xTg-AD hippocampal extractions, probed for Mgll, and phosphorylated aPKC (p-aPKC) (phosphorylation at T410/403), and re-probed for GAPDH and total aPKC (T-aPKC), relative to one of the Non-Tg samples, * P < 0.05, n = 4-6 animals/group. **(D-E)** Representative western blot images and quantitative analysis for Mgll protein levels in 6-month old WT and *Cbp*S436A hippocampal extractions, with GAPDH as a loading control and relative to one of the WT samples. * P < 0.05, n = 3 animals/genotype. **(F)** Images of human hippocampal DG sections from AD patients and their age-matched healthy controls (59-76 years) following Mgll immunohistochemistry with DAB staining. Arrows denote Mgll+ DAB stained cells. Scale bar: 50 µm. **(G)** Quantitative analysis of human hippocampal DG sections from AD patients and their age-matched healthy controls as shown in (F) using Image J analysis, relative to one of the healthy control DG sections. * P < 0.05, n = 3 sections/group. Data are represented as mean ± SEM. See also Figure S1.

Previous studies reported Mgll as a therapeutic target for AD in animal models 10,11, and showed reduced atypical protein kinase C (aPKC) expression/activity in AD patients post-mortem brain tissue 42-44. To assess how Mgll levels and aPKC expression/activity were altered during the aging process in 3xTg-AD mice, we examined Mgll protein levels and aPKC expression/activity from hippocampal tissues in both young adult (2-month-old) and middle-aged adult (9-month-old) mice. We observed similar Mgll levels between 3xTg-AD and Non-Tg hippocampal tissues at the age of 2 months. However, 3xTg-AD hippocampi exhibited significantly higher Mgll levels at the age of 9 months relative to Non-Tg (Figure 1A-B, * P < 0.05). Then, we confirmed increased Mgll signals in 12-month-old dentate gyrus (DG) tissues from 3xTg-AD animal model (Figure S1A). Thus, Mgll levels were increased in 3xTg-AD hippocampi in an age-dependent fashion. Intriguingly, when we measured aPKC expression/activity in different ages of 3xTg-AD and Non-Tg hippocampal extractions, it revealed that relative expression of total aPKC and an active form of aPKC, p-aPKC (T410/403), relative to GAPDH (as a loading control) was significantly reduced in 9-month-old 3xTg-AD hippocampi, but not 2-months-old 3xTg-AD samples (Figure 1A, C, * P < 0.05). Interestingly, our recent work identifies that an aging-dependent epigenetic pathway, involving aPKC-mediated Ser 436 phosphorylation of histone acetyltransferase CBP, is activated and is required to sustain hippocampal neuronal differentiation, maturation, and spatial memory during healthy aging from 3 to 6 months 8. Here, we further confirmed that aPKC activity was upregulated in 6-month-old hippocampal DG relative to the 3-month-old DG by immunohistochemistry analysis of p-aPKC (T410/403) expression (Figure S1B). Importantly, we observed increased Mgll expression from 6-month-old *Cbp*S436A hippocampi (Figure 1D-E, Figure S1C), arguing that Mgll expression in the hippocampus is increased when the aPKC-CBP pathway is disrupted. Finally, for the first time, we directly assessed Mgll expression in human AD brains. We examined Mgll protein levels in post-mortem human AD hippocampal DG tissues. Interestingly, AD patients' DG tissues collected from the age range of 59-76 years exhibited relatively higher and diffused Mgll signals as compared to lower and punctate Mgll signals in age-matched healthy DG tissue (Figure 1F-G, Table S1). This difference between AD and healthy controls was, however, less obvious in the older age range of 79-89 years old (Figure S1D, Table S1). Thus, these results support that aging-dependent induction of Mgll expression in the AD hippocampi is associated with a reduction of aPKC expression/activity, and that Mgll levels are upregulated following the attenuation of the aPKC-CBP pathway in phospho-null *Cbp*S436 knock-in mice during healthy aging, the activation of which sustains hippocampal neuronal differentiation and spatial memory 8.

### Aging 3xTg-AD hippocampal neuronal differentiation defects are reversed by metformin, reminiscing *Cbp*S436A deficits rescued by a Mgll inhibitor

**Figure 2:**
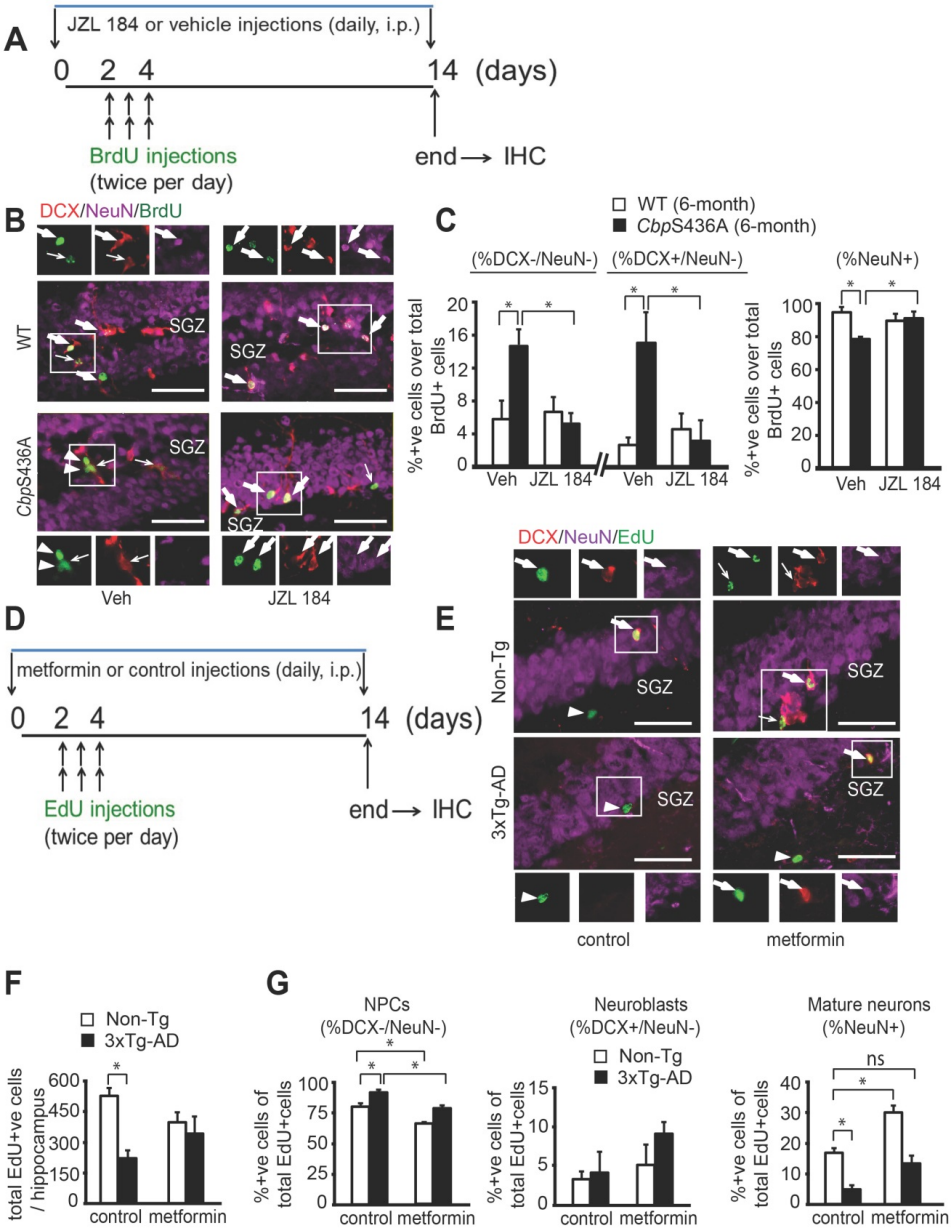
**Aging 3xTg-AD hippocampal neuronal differentiation defects are reversed by metformin, reminiscing rescue of *Cbp*S436A differentiation deficits by inhibition of Mgll activity. (A)** Schematic of the BrdU and JZL 184 injections relative to the time course of hippocampal neurogenesis analysis. JZL 184 or vehicle (as a control) was injected daily for the 14-day period and BrdU was injected at day 2 to 4 (twice per day). **(B)** Images of hippocampal DG sections from 6-month-old WT and *Cbp*S436A mice injected with JZL-184 (or vehicle) and BrdU, immunostained for BrdU (green), DCX (red), and NeuN (purple). Thick arrows denote BrdU+/ DCX+/ NeuN+ co-labelled cells; thin arrows denote BrdU+/DCX+/NeuN- cells, arrowheads denote BrdU+/DCX-/NeuN- cells. Scale bar: 50 µm. **(C)** Quantitative analysis of the percentage of NPCs (% DCX-/NeuN- over BrdU+, left panel), neuroblasts/immature neurons (% DCX+/NeuN-over BrdU+, middle panel) and mature neurons (% NeuN+ over BrdU+) in the hippocampal dentate gyrus following BrdU pulse-labelling from both WT and *Cbp*S436A mice treated with vehicle and JZL-184. Data analysis was performed using two-way ANOVA (n = 5 animals for vehicle groups, n = 4 animals for JZL 184 groups) for each cell population, * P < 0.05. **(D)** Schematic of the EdU and metformin injections relative to the time course of hippocampal neurogenesis analysis. Metformin or sterile saline (as a control) was injected daily for the 14-day period and EdU was injected at day 2 to 4 (twice per day). **(E)** Images of hippocampal DG sections from 8 to 10-month-old Non-Tg and 3xTg-AD mice treated with metformin and EdU, immunostained for EdU (green), DCX (red) and NeuN (purple). Thick arrows denote BrdU+/ DCX+/ NeuN+ co-labelled cells; thin arrows denote BrdU+/DCX+/NeuN- cells, arrowheads denote BrdU+/DCX-/NeuN- cells. Scale bar: 50 µm. **(F)** Quantification of total number of EdU+ cells throughout the extent of hippocampus. n = 3 animals/group. **(G)** Quantitative analysis of the percentage of NPCs (% DCX-/NeuN- over EdU+, left panel), neuroblasts/immature neurons (% DCX+/NeuN- over EdU+, middle panel) and mature neurons (% NeuN+ over EdU+) in the hippocampal dentate gyrus following EdU pulse-labelling from both Non-Tg and 3xTg-AD mice treated with control and metformin. Data was analysed using two-way ANOVA (n = 3 animals for Non-Tg/control, 3xTg-AD/control, and Non-Tg/metformin, n = 4 animals for 3xTg-AD/metformin) for each cell population, * P < 0.05. Data are represented as mean ± SEM.

To examine the role of Mgll in regulating hippocampal neuronal differentiation and maturation in aging *Cbp*S436A and 3xTg-AD mice, we conducted BrdU (or EdU)-chasing experiment in 6-month-old *Cbp*S436A mice and 8 to 10-month-old 3xTg-AD mice. First, to determine whether Mgll activity/expression is essential in regulating the aPKC-CBP-dependent hippocampal neurogenesis, we conducted a BrdU-chasing experiment in WT and *Cbp*S436A mice treated with either vehicle (saline containing 2.5% Tween-80 and 2% DMSO, i.p.) or JZL 184 (a Mgll activity inhibitor, 8 mg/kg, i.p., Figure 2A) for 14 days. We then performed triple immunohistochemistry for BrdU (tracing dividing cells), DCX (a marker for neuroblasts/immature neurons) and NeuN (a marker for mature neurons) in brain sections from these mice. Consistent with our previous publication 8, the proportion of mature neurons (% NeuN+ over total BrdU+ cells) was significantly reduced in 6-month-old *Cbp*S436A mice relative to their WT littermates receiving vehicle treatment (Figure 2B-C). Associated with these changes, the percentage of BrdU labelled DCX-, NeuN- NPCs (% DCX-/NeuN- over BrdU+ cells) and the proportion of BrdU labelled DCX+, NeuN- neuroblasts/immature neurons (% DCX+/NeuN- over BrdU+ cells) were significantly increased in 6-month-old *Cbp*S436A mice (Figure 2B-C). Interestingly, these hippocampal neuronal differentiation deficits in *Cbp*S436A mice were reversed by JZL 184 treatment (Figure 2B-C, two-way ANOVA for each cell population, % DCX-/NeuN-: Genotype x treatment F (1, 14) = 7.458, P = 0.0162; Genotype F (1, 14) = 3.783, P = 0.0721; Treatment F (1, 14) = 5.239, P = 0.0381; % DCX+/NeuN-: Genotype x treatment F (1, 14) = 7.56, P = 0.0157; Genotype F (1, 14) = 5.17, P = 0.0392; Treatment F (1, 14) = 4.03, P = 0.0643; % NeuN+: Genotype x treatment F (1, 14) = 10.66, P = 0.0056; Genotype F (1, 14) = 4.858, P = 0.0448; Treatment F (1, 14) = 2.889, P = 0.1113, n = 18, Tukey's post-hoc test, * P < 0.05). Thus, this data argues that activation of the aPKC-CBP pathway represses Mgll expression/activity to sustain hippocampal neuronal differentiation in mature adult (6-month-old) hippocampi.

To further ask whether the increased Mgll levels in aging 3xTg-AD hippocampi could cause similar deficits in hippocampal neuronal differentiation as shown in mature adult *Cbp*S436A mice, we performed similar experiments in 8 to 10-month-old Non-Tg and 3xTg-AD mice. EdU-chasing experiment (Figure 2D) revealed that the total number of EdU+ cells throughout the extent of hippocampus were significantly reduced in the 3xTg-AD mice (Figure 2E-F), suggesting a reduced pool of dividing NPCs in the 3xTg-AD hippocampi. These results were consistent with previous findings 41. Importantly, we observed that the proportion of mature neurons (% NeuN+ over total EdU+ cells) was significantly reduced and the percentage of EdU labelled DCX-, NeuN- NPCs (% DCX-/NeuN- over BrdU+ cells) was increased in 3xTg-AD mice (Figure 2E, 2G).

Since metformin, an FDA-approved drug, can activate the aPKC-*Cbp* pathway to promote NPC neuronal differentiation, we asked whether metformin treatment in 3xTg-AD mice could rescue the observed deficits in hippocampal neurogenesis. For this, we treated 8 to10-month-old Non-Tg and 3xTg-AD mice with metformin (daily, i.p. 200 mg/kg) starting 2 days before EdU injections (twice per day for 3 days, i.p. 50 mg/kg) for 14 days, and then sacrificed them for immunohistochemistry (Figure 2D-G). We found that metformin treatment erased the difference between 3xTg-AD and Non-Tg hippocampi in terms of total number of EdU+ cells (Figure 2E-G, two-way ANOVA: Genotype x treatment F (1, 8) = 1.841, P = 0.21; Genotype F (1, 8) =14.2, P = 0.005; Treatment F (1, 8) =0, P > 0.99, n = 12, Tukey's post-hoc test, *P < 0.05). In addition, metformin treatment rescued the reduced mature neuronal population (Figure 2E,G, % NeuN+ over EdU+ cells, two-way ANOVA: Genotype x treatment F (1, 9) = 0.7175, P = 0.4189; Genotype F (1, 9) = 32.44, P = 0.0003; Treatment F (1, 9) = 16.57, P = 0.0028, n = 13, Tukey's post-hoc test, * P < 0.05), as well as, reversed the increased NPC population (Figure 2E-G, % DCX-/NeuN- over EdU+ cells, two-way ANOVA: Genotype x treatment F (1, 9) = 0.0746, P = 0.7908; Genotype F (1, 9) = 29.90, P = 0.0004; Treatment F (1, 9) = 39.14, P = 0.0001, n = 13, Tukey's post-hoc test, * P < 0.05) in 3xTg-AD mice back to the basal level (Non-Tg/control). Together, our data shows that 8 to 10-month-old 3xTg-AD hippocampi, exhibiting reduced aPKC activity and increased Mgll levels, replicates mature adult *Cbp*S436A hippocampal neuronal differentiation defects, which can be rescued by metformin treatment.

### Aging 3xTg-AD spatial memory deficits are prevented by Mgll repression through metformin-stimulated aPKC-CBP signaling

**Figure 3:**
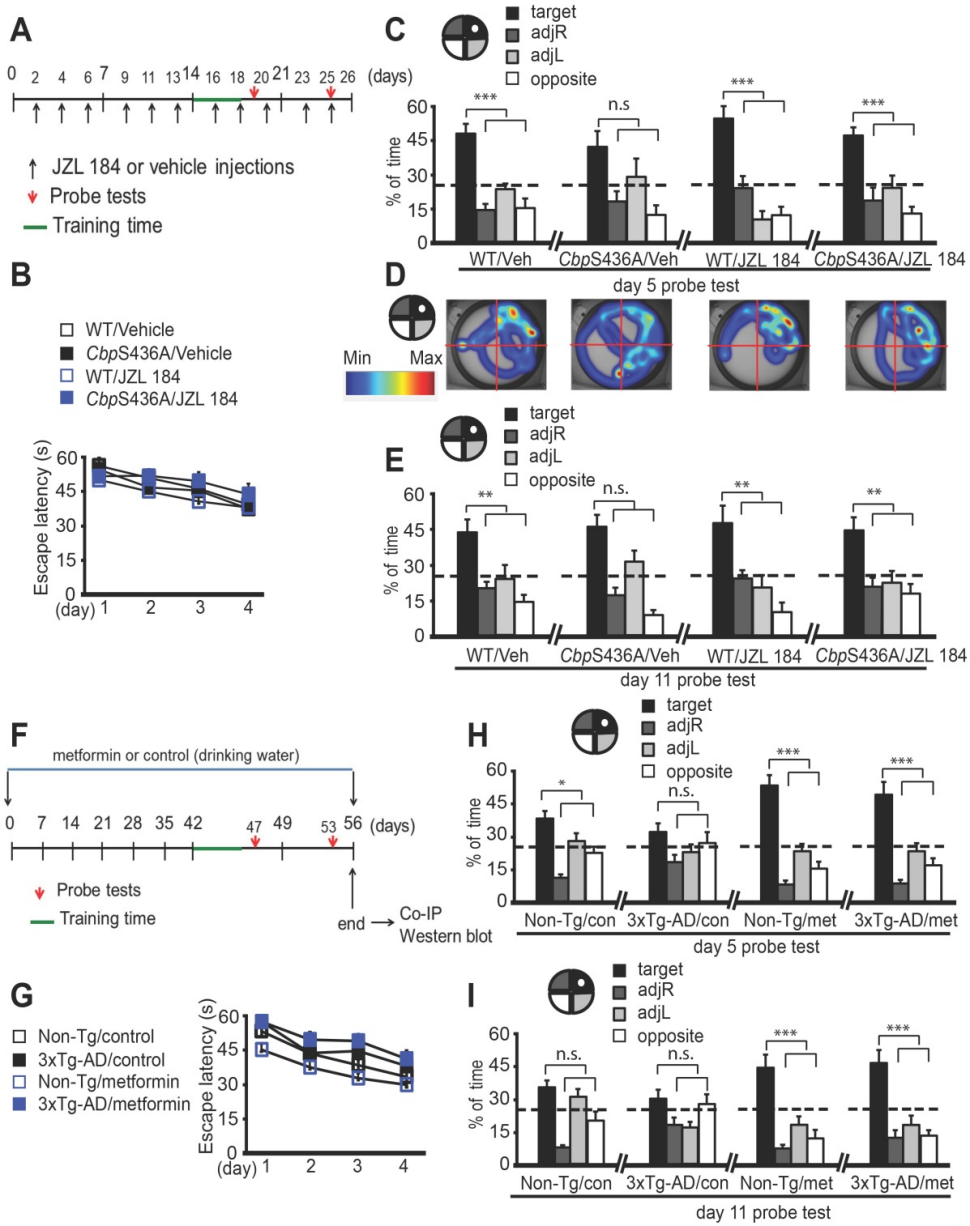
**Metformin prevents 3xTg-AD spatial memory decline, reminiscent of JZL 184 rescuing mature adult *Cbp*S436A spatial memory deficits**. **(A)** Schematic of the JZL 184 injections relative to the time course of training and testing protocols for the Morris water maze (MWM) experiment. JZL 184 or vehicle (as a control) was injected every 2-3 days for the 25-day period. **(B)** Acquisition of the platform location in the MWM task is across 4 days of training with latency to reach the platform as a measurement of learning. WT and *Cbp*S436A mice receiving vehicle and JZL 184 treatments behaved in a similar manner in the initial acquisition of the task (n=10-11 animals/group). **(C)** After acquisition, the platform was removed and mice were given a 60 s probe trial. The percentage of time spent in the 4 quadrant zones was analyzed at day 5 as a measurement of short-term memory, *** P < 0.001. **(D)** Representative heat maps from each group shown in C. **(E)** The percentage of time spent in the 4 quadrant zones was analysed at day 11 as a measurement of long-term memory, ** P < 0.01. **(F)** Schematic of metformin treatment relative to the time course of training and testing protocols for the MWM experiment. Metformin or sterile water (as a control) was delivered through drinking water for the 56-day period. **(G)** Acquisition of the platform location in the MWM task is across 4 days of training with latency to reach the platform as a measurement of learning. Non-Tg and 3xTg-AD mice receiving control and metformin treatments behaved in a similar manner in the initial acquisition of the task (n = 13-23 animals/group). **(H-I)** After acquisition, the platform was removed and mice were given a 60 s probe trial. The percentage of time spent in 4 quadrant zones was analysed at day 5 as a measurement of short-term memory, *** P < 0.001, * P<0.05. The percentage of time spent in 4 quadrant zones was analysed at day 11 as a measurement of long-term memory, ***P<0.001. Data are represented as mean±SEM. See also Figure S2.

To ask whether inhibition of Mgll activity can rescue spatial memory deficits in 6-month-old *Cbp*S436A mice reported previously 8, we performed a hidden-platform version of Morris Water Maze (MWM) task in 6-month-old *Cbp*S436A mice and their WT littermates treated with vehicle or JZL 184 (a Mgll activity inhibitor) (Figure 3A). We showed that WT and *Cbp*S436A mice had a comparable learning curve over 4 days (Figure 3B, two-way ANOVA: Group x training day F (9, 152) = 0.3530, P = 0.9551; group F (3, 152) = 1.827, P = 0.1445; training day F (3, 152) = 9.374, P < 0.0001; n = 42). Following the 4-day training session, probe tests were conducted on day 5 and day 11 by removing the platform from the swimming pool. On day 5, 24 h after last training, *Cbp*S436A mice receiving vehicle treatment did not show a specific preference to the target quadrant (one way ANOVA, *Cbp*S436A/ vehicle: F (3, 40) = 4.679, P = 0.0068, for the target quadrant relative to all other quadrants, n.s. Dunnett's post-hoc test, P > 0.05, n = 11 animals). However, WT mice receiving the same vehicle treatment spent significantly more time in the target quadrant than the other three quadrants (Figure 3C-D, one ay ANOVA, WT/vehicle: F (3, 40) = 21.58, P < 0.0001, target quadrant > all other quadrants, Dunnett's post-hoc test, *** P < 0.001, n = 11 animals). This short-term memory deficit in 6-month-old *Cbp*S436A mice was not observed in our previously published work 8, in which an elongated training time (7-day training session) was used, possibly overriding the short-term memory deficit observed in the current study. Interestingly, this short-term memory deficit was reversed in the *Cbp*S436A mice treated with JZL 184, that spent significantly more time in the target quadrant (Figure 3C-D, one way ANOVA, WT/ JZL 184: F (3, 36) = 9.928, P < 0.0001, target quadrant > all other quadrants, Dunnett's post-hoc test, *** P < 0.001, n = 10 animals; *Cbp*S436A/ JZL 184: F (3, 36) = 16.04, P < 0.0001, target quadrant > all other quadrants, Dunnett's post-hoc test, *** P < 0.001, n = 10 animals). Similarly, in the late probe test on day 11 (7 d after training), *Cbp*S436A mice receiving JZL 184 treatment were able to retain the long-term spatial memory that was lost in *Cbp*S436A mice receiving vehicle treatment (Figure 3E, one way ANOVA, WT/vehicle: F (3, 40) = 7.131, P=0.0006, target quadrant > all other quadrants, Dunnett's post-hoc test, ** P < 0.01, n = 11 animals; *Cbp*S436A/vehicle: F (3, 40) = 13.81, P < 0.0001, for the target quadrant relative to all other quadrants, n.s. Dunnett's post-hoc test, P > 0.05, n = 11 animals; WT/JZL 184: F (3, 36) = 4.991, P < 0.0054, target quadrant > all other quadrants, Dunnett's post-hoc test, ** P < 0.01, n = 10 animals; *Cbp*S436A /JZL 184: F (3, 36)=7.306, P = 0.0006, target quadrant > all other quadrants, Dunnett's post-hoc test, ** P < 0.01, n = 10 animals). In addition, JZL 184 treatment did not significantly alter mean velocity and distance traveled in WT and *Cbp*S436A mice, respectively (Figure S2A- B). Together, these data suggest that Mgll repression is essential to form spatial memory in mature adults where the aPKC-CBP pathway is fully active.

We subsequently used the same paradigm of MWM task to show that 3xTg-AD mice at the age of 8-10 months had a similar learning curve over 4 days as age-matched Non-Tg mice (Figure 3F-G, two-way ANOVA: Group x training day F (9, 244)=0.3301, P = 0.9645; Group F (3, 244)=11.72, P<0.0001; Training day F (3, 244)=22.4, P<0.0001, n=65). However, at the day 5 probe test, 3xTg-AD mice showed impaired short- term spatial memory, manifesting no significant preference to the target quadrant (Figure 3H, one way ANOVA, 3xTg-AD vehicle: F (3, 52)=2.392, P=0.0791, for the target quadrant relative to all other quadrants, n.s. Dunnett's post-hoc test, P>0.05, n=14 animals). On the other hand, Non-Tg mice spent significantly more time in the target quadrant (Figure 3H, one way ANOVA, Non-Tg vehicle: F (3, 88)=20.1, P < 0.0001, target quadrant > all other quadrants, Dunnett's post- hoc test, *P<0.05, n=23 animals). Interestingly, at the day 11 probe test for long term-spatial memory, both Non-Tg and 3xTg-AD mice did not spend significantly more time in the target quadrant (Figure 3I, one way ANOVA, Non-Tg vehicle: F (3, 88) = 16.49, P < 0.0001, for the target quadrant relative to all other quadrants, n.s. Dunnett's post-hoc test, P > 0.05, n = 23 animals; 3xTg-AD vehicle: F (3, 52) = 3.704, P = 0.0172, for the target quadrant relative to all other quadrants, n.s. Dunnett's post-hoc test, P > 0.05, n = 14 animals). To ask whether metformin treatment could rescue the spatial memory deficits in 3xTg-AD mice by reducing Mgll expression through reactivating the impaired aPKC-CBP pathway, we treated both Non-Tg and 3xTg-AD mice with metformin through drinking water (4 mg/mL) starting at the age of 7-8 months for a period of 6 weeks and throughout the MWM task experiment (Figure 3F). Excitingly, metformin treatment rescued the short-term memory deficits in 3xTg-AD mice (Figure 3H, one way ANOVA, Non-Tg metformin: F (3, 48)=36.36, P<0.0001, target quadrant > all other quadrants, Dunnett's post-hoc test , *** P < 0.001, n=13 animals; 3xTg-AD metformin: F (3, 56)= 27.93, P<0.0001, target quadrant > all other quadrants, Dunnett's post-hoc test, *** P < 0.001, n = 15 animals), and significantly improved long-term spatial memory in both Non-Tg and 3xTg-AD mice (Figure 3I, one way ANOVA, Non-Tg metformin: F (3, 48) = 17.21, P < 0.0001, target quadrant > all other quadrants, Dunnett's post-hoc test, *** P < 0.001, n = 13 animals; 3xTg-AD metformin: F (3, 56) = 14.76, P < 0.0001, target quadrant > all other quadrants, Dunnett's post-hoc test, *** P < 0.001, n = 15 animals). In addition, both Non-Tg and 3xTg-AD mice treated with control and metformin showed normal mean velocity and distance traveled (Figure S2C-D), making it unlikely that the spatial memory deficits and rescue effects are attributable to non-specific motor function.

**Figure 4:**
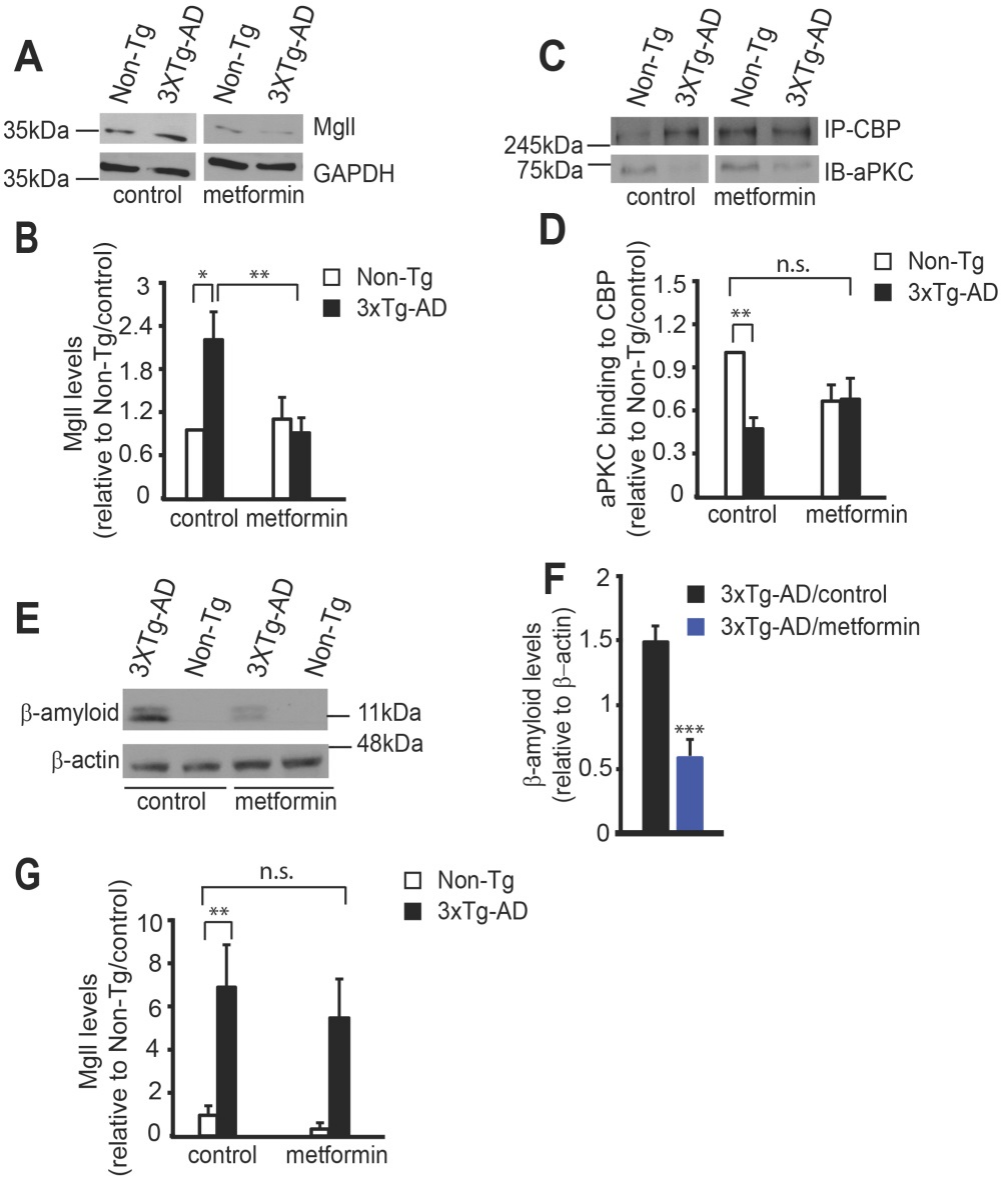
**Metformin reactivates the aPKC-CBP pathway to repress Mgll expression. (A-B)** Representative western blot images (A) and quantitative analysis (B) of total protein lysates from Non-Tg and 3xTg-AD mice receiving control and metformin treatment at the end of behavioural experiments, probed for Mgll and re-probed for GAPDH (a loading control). * P < 0.05, ** P < 0.01, n = 6-7 animals/group. **(C-D)** Representative western blot images (C) and quantitative analysis (D) of association of aPKC with CBP after immunoprecipitation. The hippocampal lysates extracted from Non-Tg and 3xTg-AD mice at the end of behavioural experiments were immunoprecipitated with a CBP antibody, washed and then blotted with anti-aPKC and anti-CBP antibodies. ** P < 0.01, n = 6 animals/group. n.s. no-significance. **(E-F)** Representative western blot images (E) and quantitative analysis (F) of total protein lysates from Non-Tg and 3xTg-AD mice receiving control and metformin treatment at the end of behavioural experiments, probed for β-amyloid and re-probed for β-actin (a loading control). Student's t-test was performed for n = 4 animals/group. *** P < 0.001. **(G)** Quantitative PCR (qPCR) for Mgll mRNA from circulating blood cells collected at the end of behavioural experiments. ** P < 0.01, n = 9-13 animals/group. n.s. no significance. Data are represented as mean ± SEM. See also Figure S3.

At the end of behavioural experiments, we assessed Mgll expression and association of aPKC with CBP protein in isolated hippocampal extractions. We observed that metformin treatment successfully reversed the increased Mgll expression in 3xTg-AD mice back to the basal level (Non-Tg/control) (Figure 4A-B, two-way ANOVA: Genotype x treatment F (1, 22) = 7.333, P = 0.0128; Genotype F (1, 22) = 3.423, P = 0.0778; Treatment F (1, 22) = 5.151, P = 0.0334, n = 6-7, Tukey's post-hoc test, *P<0.05, **P<0.01). In addition, co-immunoprecipitation experiment showed that reduced aPKC binding to CBP protein in 3xTg-AD hippocampi was rescued by metformin treatment (Figure 4C-D, two-way ANOVA: Genotype x treatment F (1, 20) = 6.698, P = 0.0176; Genotype F (1, 20) = 7.689, P = 0.0117; Treatment F (1, 20) = 0.7636, P = 0.3926, n = 6, Tukey's post-hoc test, ** P < 0.01). Thus, the data strongly argue that 3xTg-AD mice show increased Mgll levels due to an impaired aPKC-CBP pathway in the aging hippocampi, associated with spatial memory deficits. Importantly, metformin treatment is able to reactivate this impaired aPKC-CBP signaling to reduce Mgll expression, thus, preventing the deficits in hippocampal spatial memory.

### Mgll serves as a biomarker for identifying potential metformin-responsive AD patients

One of the neuropathological hallmarks of AD is β-amyloid (Aβ) accumulation. To examine whether metformin treatment can reduce the Aβ burden in the brain, we performed a western blot analysis using hippocampal extractions isolated after behavioural tests and showed that metformin treatment significantly reduced Aβ accumulation in the 3xTg-AD hippocampi (Figure 4E-F, Student's t-test, *** P < 0.001). In addition, immunohistochemistry analysis revealed that metformin significantly reduced intracellular Aβ accumulation in the DG of 8-month-old 3xTg-AD hippocampi, an early sign of AD-associated neuropathology (Figure S3A-B).

Lastly, to assess whether Mgll expression in peripheral blood can be used as a biomarker for early detection of AD and as a risk factor for assessing effective treatment of metformin, we conducted RT-PCR to assess *Mgll* mRNA expression from peripheral blood at the end of behavioural experiments. We found that 3xTg-AD/control mice exhibited a robust increase in *Mgll* mRNA levels relative to Non-Tg/control mice (Figure 4G), reminiscent of 3xTg-AD hippocampal Mgll protein levels (Figure 4A). Intriguingly, metformin treatment in 3xTg-AD mice reduced *Mgll* mRNA expression, showing a non-significant change relative to Non-Tg/control mice (Figure 4G, two-way ANOVA: Genotype x treatment F (1, 39) = 0.1481, P = 0.70; Genotype F (1, 39) = 18.63, P = 0.0001; Treatment F (1, 39) = 0.6555, P = 0.42, n = 43, Tukey's post-hoc test, ** P < 0.01). Together, these findings suggest that Mgll could be used as an early biomarker for identifying potential metformin-responsive AD patients.

### Metformin-induced aPKC-CBP signaling directly represses Mgll expression to promote adult NPC neuronal differentiation

**Figure 5:**
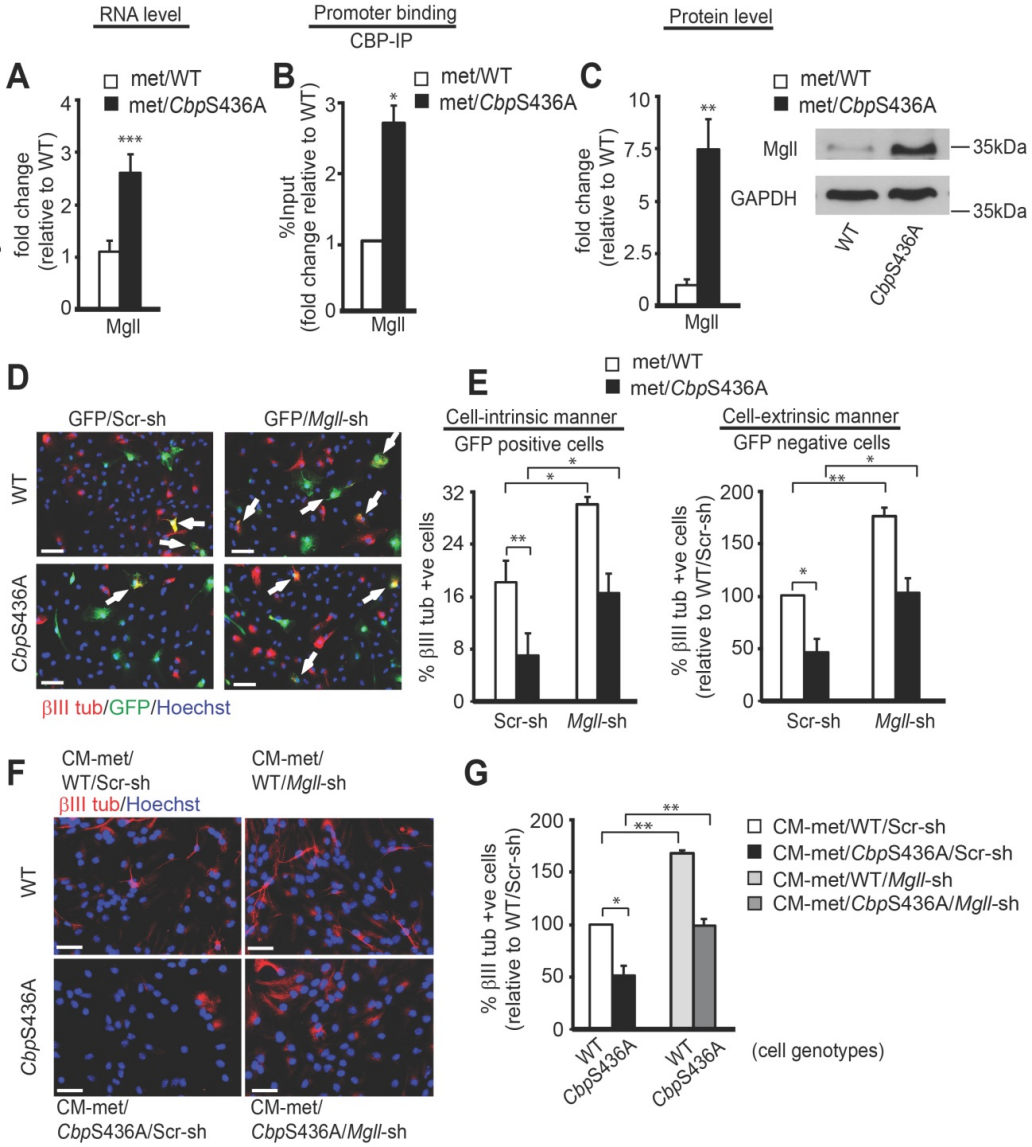
**Metformin-induced aPKC-CBP signaling represses Mgll expression to promote adult NPC neuronal differentiation. (A)** qPCR for Mgll mRNA from differentiating WT and *Cbp*S436A NPCs in the presence of metformin (met, 1 μM). *** P < 0.001 (n = 4 animals/group). **(B)** ChIP-qPCR analysis for CBP binding at Mgll promoter in differentiating WT and *Cbp*S436A NPCs in the presence of metformin (1 μM), relative to corresponding WT NPC samples. * P < 0.05 (n = 3 animals/group). **(C)** Representative western blot images and quantitative analysis for Mgll protein levels in differentiating P2 WT and *Cbp*S436A NPCs in the presence of metformin (1 μM) for 7 days, with GAPDH as a loading control and relative to one of WT NPC samples. ** P < 0.01 (n = 4 animals/group). **(D-E)** Immunofluorescent images (D) and quantitative analysis (E) of the percentage of βIII tubulin+ (red)/GFP+ (green) co-labeled neurons out of total GFP+ cells (left panel) and the percentage of βIII tubulin+(red)/ GFP- cells out of total GFP- cells (right panel) from differentiating P2 WT and *Cbp*S436A NPCs following transfection with Scr or Mgll shRNAs, together with an eGFP plasmid, in the presence of metformin (1 μM) for 7 days. Data were analysed by two-way ANOVA, n = 3-4 animals/group, * P<0.05, ** P < 0.01; Scale bar: 100 μm. **(F-G)** Immunofluorescent images (F) and quantitative analysis (G) of the percentage of βIII tubulin+ neurons out of total live P2 WT and *Cbp*S436A NPCs treated with 50% conditioned medium (CM) from differentiating P2 NPCs transfected with Scr or Mgll shRNAs. Data were analysed by two-way ANOVA, n = 3 animals/group, * P < 0.05, ** P < 0.01. Scale bar: 100 μm. Data are represented as mean ± SEM. See also Figure S4. Scr-sh: Scramble shRNA, Mgll-sh: Mgll shRNAs; CM-WT/Scr-sh: CM from WT NPCs transfected with Scr-sh; CM-WT/Mgll-sh: CM from WT NPCs transfected with Mgll-sh; CM-*Cbp*S436A/Scr-sh: CM from *Cbp*S436A NPCs transfected with Scr-sh; CM-*Cbp*S436A/Mgll-sh: CM from *Cbp*S436A NPCs transfected with Mgll-sh.

To support a clinical protocol that *Mgll* mRNA levels in peripheral blood can be used as an early biomarker for identifying potential metformin- responsive AD patients, we further delineated how metformin regulates *Mgll* gene expression through the aPKC-CBP pathway. In this regard, we previously showed that metformin promotes adult subventricular zone (SVZ) NPC neuronal differentiation in culture by stimulating the aPKC-CBP pathway 24. Here, we used the same SVZ NPC culture model to identify the molecular targets through which metformin acts to promote the neuronal differentiation of adult NPCs. Hereby, we conducted an RNA-seq analysis using differentiating WT and *Cbp*S436A NPCs cultured in the presence of metformin to identify its downstream targets. In total, 48 genes were dysregulated in *Cbp*S436A NPCs (Table S2), including upregulated expression of *Mgll* gene. We subsequently confirmed that *Mgll* mRNA and protein levels were aberrantly upregulated in differentiating *Cbp*S436A NPCs (Figure 5A, C, Student's t-test, *** P < 0.001, ** P < 0.01) in the presence of metformin. To ask whether CBP directly regulates *Mgll* expression, we performed a ChIP assay using an anti-CBP antibody to pull down *Mgll* promoter and showed that *Cbp*S436A has a significantly higher binding ability at the *Mgll* promoter in differentiating NPCs (Figure 5B and Figure S4A, Student's t-test, * P < 0.05).

To assess whether increased Mgll levels in *Cbp*S436A NPCs were responsible for their neuronal differentiation deficit, thus lacking the response to metformin treatment 24, we designed two *Mgll* shRNAs (*Mgll* shRNA 1, *Mgll* shRNA 2), each targeting a different region of the *Mgll* transcript, to knock down Mgll expression. We first confirmed the knockdown efficacy of these *Mgll* shRNAs using NIH3T3 cells. We showed that the combination of *Mgll* shRNA 1 and 2 consistently and efficiently reduced endogenous Mgll protein levels in NIH3T3 cells (Figure S4B-D), and thus chose this formula (shRNA1+2) to ask what roles Mgll played in mediating the aPKC-CBP pathway to regulate adult SVZ NPC neuronal differentiation.

We co-transfected passage 2 (P2) WT and *Cbp*S436A SVZ NPCs with an eGFP plasmid and either *Mgll* shRNAs (1+2) or Scrambled (Scr) shRNA and analyzed neuronal differentiation when the NPCs were directed to differentiate in the presence of metformin (1 μM) for 7 days. Neuronal differentiation was assessed by immunocytochemistry for GFP and βIII tubulin, a newborn neuron marker. We observed that knockdown of Mgll significantly increased the proportion of βIII tubulin+ neurons in both WT and *Cbp*S436A NPCs in the presence of metformin (Figure 5D-E). Importantly, Mgll knockdown rescued the reduced percentage of βIII tubulin+ neurons produced from *Cbp*S436A NPCs back to the basal levels observed in WT NPCs in the presence of metformin (Figure 5D-E, two-way ANOVA: Genotype x treatment F (1, 8) = 0.0642, P = 0.8064; Treatment F (1, 8) = 21.56, P = 0.0017; Genotype F (1, 8) = 28.02, P = 0.0007, n = 12, Tukey's post-hoc test, ** P < 0.01, * P < 0.05). These results indicate that *Mgll* knockdown rescues neuronal differentiation deficits of *Cbp*S436A NPC in a cell-intrinsic manner.

Intriguingly, quantification of the percentage of βIII tubulin+/GFP- neurons (generated from non- transfected NPCs) out of total live cells in the same set of experiments revealed a similar rescuing effect upon Mgll knockdown (Figure 5D-E, two-way ANOVA: Genotype x treatment F (1, 12)=1.230, P=0.2891; Treatment F (1, 12)=39.05, P<0.0001; Genotype F (1, 12)= 40.37, P<0.0001, n=16, Tukey's post-hoc test, **P< 0.01, *P < 0.05). These results support that Mgll knockdown additionally acts in a cell-extrinsic manner to promote adjacent NPC neuronal differentiation. To further confirm this cell-extrinsic effect, we collected conditioned media (CM) from differentiating P2 WT and *Cbp*S436A NPCs transfected with Scr or *Mgll* shRNAs and treated a fresh set of differentiating P2 NPCs with 50% of this CM for 7 days *in vitro* (DIV) in the presence of metformin (1 μM), without direct Mgll knockdown. Immunocytochemical analysis of the percentage of βIII tubulin+ neurons generated from the CM-treated NPCs showed that the neuronal differentiation defect in the P2 *Cbp*S436A NPCs was rescued upon treatment with CM from *Mgll*-shRNA transfected *Cbp*S436A NPCs (Figure 5F-G, two-way ANOVA (Genotype x treatment F (1, 8) = 0.1839, P = 0.6794; Treatment F (1, 8)=44.75, P = 0.0002; Genotype F (1, 8) = 29.50, P = 0.0006, n = 12, Tukey's post-hoc test,** P < 0.01, * P < 0.05), in a similar manner as the P2 *Cbp*S436A NPCs that were directly transfected with *Mgll* shRNA. These results confirm the cell-extrinsic effects of Mgll knockdown in rescuing *Cbp*S436A NPC neuronal differentiation deficits. In addition, these culture data fully support our *in vivo* neurogenesis results where increased Mgll levels in *Cbp*S436A and 3xTg-AD hippocampi impaired appropriate hippocampal neuronal differentiation and reduction of Mgll activity/levels was able to reverse the differentiation deficits (Figure 2).

### 3xTg-AD NPCs exhibit reduced aPKC activity, increased Mgll levels, and replicate the neuronal differentiation and proliferation defects of *Cbp*S436A NPCs

**Figure 6:**
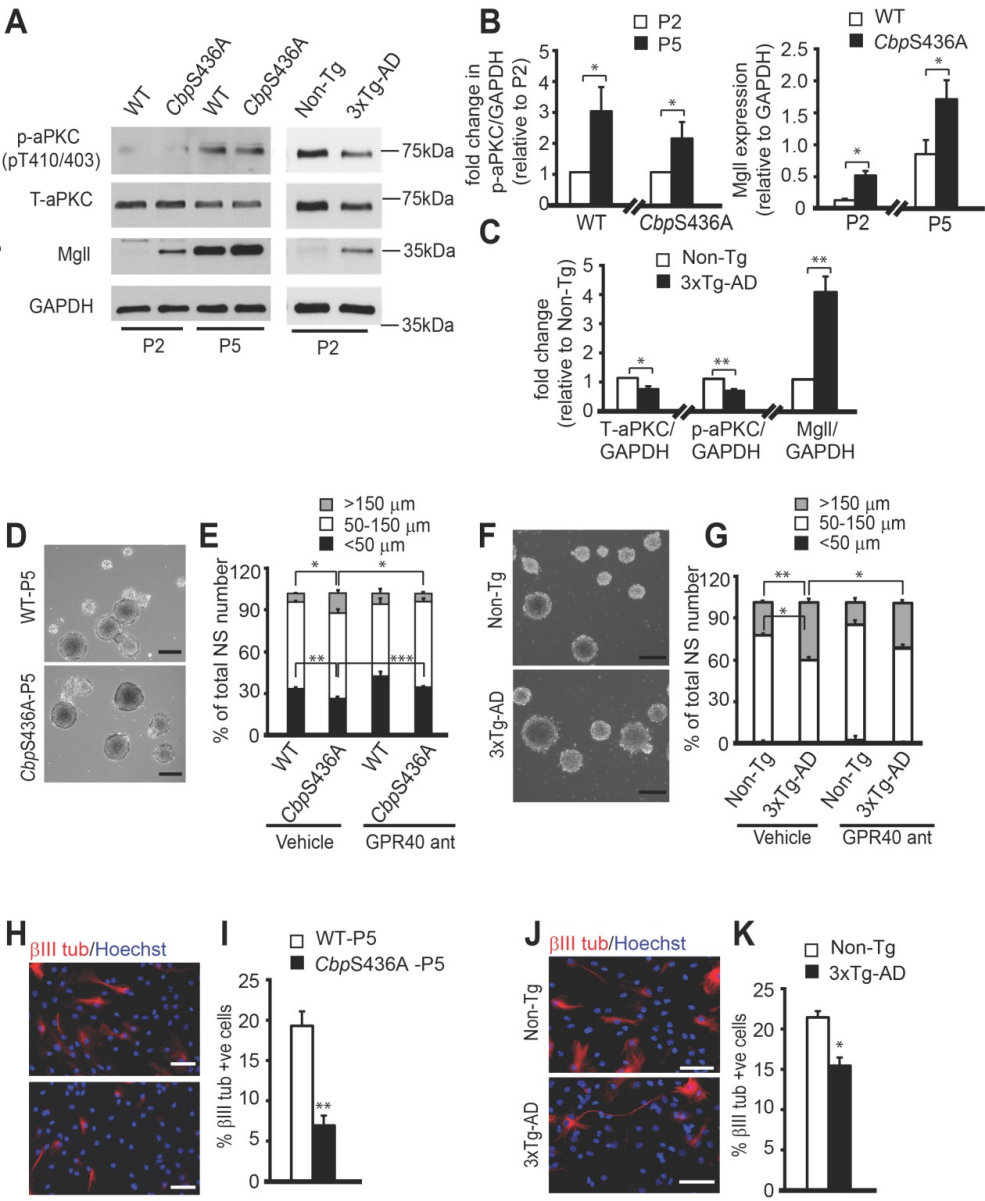
**3xTg-AD NPCs exhibit reduced aPKC activity, increased Mgll levels, and replicate the neuronal differentiation and proliferation defects of *Cbp*S436A NPCs. (A)** Representative western blot images of total protein lysates from proliferating P2 (early passage) and P5 (late passage) WT and *Cbp*S436A NPCs (left panel) and P2 Non-Tg and 3xTg-AD NPCs (right panel), probed for Mgll and phosphorylated aPKC (p-aPKC) (phosphorylation at T410/403), and re-probed for GAPDH and total-aPKC (T-aPKC) as loading controls. **(B)** Quantitative analysis of aPKC activity, measured as p-aPKC (phosphorylation at T410/403) protein expression relative to GAPDH, and Mgll expression relative to GAPDH in proliferating P2 and P5 WT and *Cbp*S436A NPCs. Data was normalized to corresponding P2 NPCs for each genotype and analyzed by Student's t-test, n = 4 animals/group, * P < 0.05. **(C)** Quantitative analysis of T-aPKC, p-aPKC and Mgll protein levels relative to GAPDH in 3xTg-AD and Non-Tg NPCs. n = 5 animals/group, * P < 0.05, ** P < 0.01. **(D)** Representative bright-field images of P5 WT and *Cbp*S436A neurospheres. Scale bar: 200 µm. **(E)** Quantitative analysis of the percentage of small (< 50 µm), mid-sized (50-150 µm), and large (> 150 µm) neurospheres generated from WT and *Cbp*S436A P5 NPCs, treated with vehicle (0.01% DMSO) and GPR40 antagonist (100 nM DC260126). Data analysis was performed using two-way ANOVA for each size bracket, n = 3 animals/group * P < 0.05, ** P < 0.01. *** P < 0.001. **(F)** Representative bright-field images of P2 Non-Tg and 3xTg-AD neurospheres. Scale bar: 200 µm. **(G)** Quantitative analysis of the percentage of small (< 50 µm), mid-sized (50-150 µm), and large (> 150 μm) neurospheres generated from P2 Non-Tg and 3xTg-AD NPCs, treated with vehicle (0.01% DMSO) and GPR40 antagonist (100 nM DC260126). Statistical analysis was performed using two-way ANOVA for each size bracket, n = 3 animals per group, * P < 0.05, ** P < 0.01. **(H, J)** Representative immunofluorescent images of differentiating WT and *Cbp*S436A P5 NPCs (H) and of differentiating Non-Tg and 3xTg-AD NPCs (J), immunostained for βIII tubulin (red) and Hoechst (blue). Scale bar: 50 μm (H), 25 μm (J). **(I-K)** Quantitative analysis of the percentage of βIII tubulin+ neurons generated from WT and *Cbp*S436A P5 NPCs (I) and from P2 Non-Tg and 3xTg-AD NPCs (K) 7 days upon differentiation. n = 3 animals/group, * P < 0.05, ** P < 0.01. Data are represented as mean ± SEM. See also Figure S5.

We next sought to develop a culture model to study the role of Mgll in regulating 3xTg-AD and *Cbp*S436A NPC function. In this regard, we observed that both 6-8 weeks WT and *Cbp*S436A passage 5 (P5) NPCs exhibited elevated aPKC activity compared to their respective P2 counterparts (Figure 6A-B, Student's t-test, * P < 0.05), measured by levels of an active form of aPKC, p-aPKC (T410/403). The total aPKC levels were not changed by continued passaging (Figure S5). These results suggested that continued passaging can intrinsically activate the aPKC-CBP pathway in the P5 NPCs. Interestingly, both WT and *Cbp*S436A P5 NPCs exhibited higher Mgll levels than their P2 counterparts despite enhanced aPKC activity (Figure 6A-B, Student's t-test, * P < 0.05), implicating that other mechanisms are involved to enhance Mgll protein levels through the continued passaging. The raised Mgll levels in P5 NPCs argue strongly for their key role in regulating P5 NPC function. Importantly, Mgll levels in the P5 *Cbp*S436A NPCs were significantly higher than those in the P5 WT NPCs (Figure 6A-B). These findings demonstrate that continued passaging of NPCs intrinsically activates the aPKC-CBP pathway to repress Mgll expression, as shown *in vivo* during aging. Thus, it represents an additional culture model to study the role of this pathway in regulating NPC function.

Intriguingly, previous studies have reported decreased aPKC activation in post-mortem AD brains 42-44 and have identified Mgll as a therapeutic target in animal models of AD 10,11. These findings along with our initial results, prompted us to ask if aPKC-CBP mediated Mgll repression was impaired in young adult AD NPCs, an early sign of pathological aging in NPC function. To test this, we assessed the status of aPKC activity and Mgll levels in P2 NPCs derived from 6-8 weeks 3xTg-AD mice. We observed a significant decrease in both T-aPKC and p-aPKC (phosphorylation at T410/403) levels in 3xTg-AD NPCs relative to Non-Tg NPCs, associated with elevated Mgll expression (Figure 6A, C). These findings confirmed previous reports showing impaired aPKC expression/activity and Mgll upregulation in AD, supporting that SVZ neurosphere culture is a suitable model to study the impaired pathway in the context of AD.

Following these studies, we used the P5 *Cbp*S436A NPCs and P2 3xTg-AD NPCs to examine the role of the aPKC-CBP mediated Mgll repression in regulating NPC proliferation versus differentiation. NPC proliferation was assessed by measuring the neurosphere diameter, while neuronal differentiation was assessed by quantifying the percentage of βIII tubulin+ neurons produced from the same group of NPCs when directed to differentiate. We found that P5 *Cbp*S436A NPCs and P2 3xTg-AD NPCs formed a larger size of neurospheres than their respective controls: P5 WT NPCs and P2 Non-Tg NPCs (Figure 6D-G).

Since ARA-GPR40 signaling is known to promote primate NPC proliferation 45, we asked whether increased ARA-GPR40 signaling, due to elevated Mgll levels in P5 *Cbp*S436A and P2 3xTg-AD NPCs, is responsible for the observed enhanced proliferation. To test this, we treated these NPCs with a potent GPR40 antagonist, DC260126, and showed that 100 nM DC260126 was able to reverse the increased size of neurospheres in P5 *Cbp*S436A NPCs (Figure 6D-E, two-way ANOVA for each size bracket: < 50 μm: Genotype x treatment F (1, 8) = 1.161, P = 0.3126; Genotype F (1, 8) = 46.58, P = 0.0001; Treatment F (1, 8) = 66.82, P < 0.0001; 50-150 μm: Genotype x treatment F (1, 8) = 3.274, P = 0.1080; Genotype F (1, 8) = 4.718, P = 0.0616; Treatment F (1, 8) = 6.596, P = 0.0332; > 150 μm: Genotype x treatment F (1, 8) = 6.238, P = 0.0371; Genotype F (1, 8) = 4.135, P = 0.0764; Treatment F (1, 8) = 6.094, P = 0.0388, n = 12, Tukey's post-hoc test, * P < 0.05, ** P < 0.01. *** P < 0.001) and P2 3xTg-AD NPCs (Figure 6F-G, two-way ANOVA for each size bracket: < 50 μm: Genotype x treatment F (1, 8) = 0.3945, P = 0.5640; Genotype F (1, 8) = 0.5295, P = 0.5071; Treatment F (1, 8) = 1.127, P = 0.3482; 50-150 μm: Genotype x treatment F (1, 8) = 0.3917, P = 0.5653; Genotype F (1, 8) = 51.96, P = 0.002; Treatment F (1, 8) = 9.647, P = 0.036; > 150 μm: Genotype x treatment F (1, 8) = 0.3010, P = 0.6124; Genotype F (1, 8) = 198.4, P = 0.0001; Treatment F (1, 8) = 46.24, P = 0.0024, n = 12, Tukey's post-hoc test,* P < 0.05, ** P < 0.01) back to basal levels observed in the controls but did not have significant effects on WT and Non-Tg NPCs (Figure 6D-G). These results support that elevated Mgll levels in the P5 *Cbp*S436A NPCs and P2 3xTg-AD NPCs promote their proliferation by activating ARA-GPR40 signaling.

**Figure 7:**
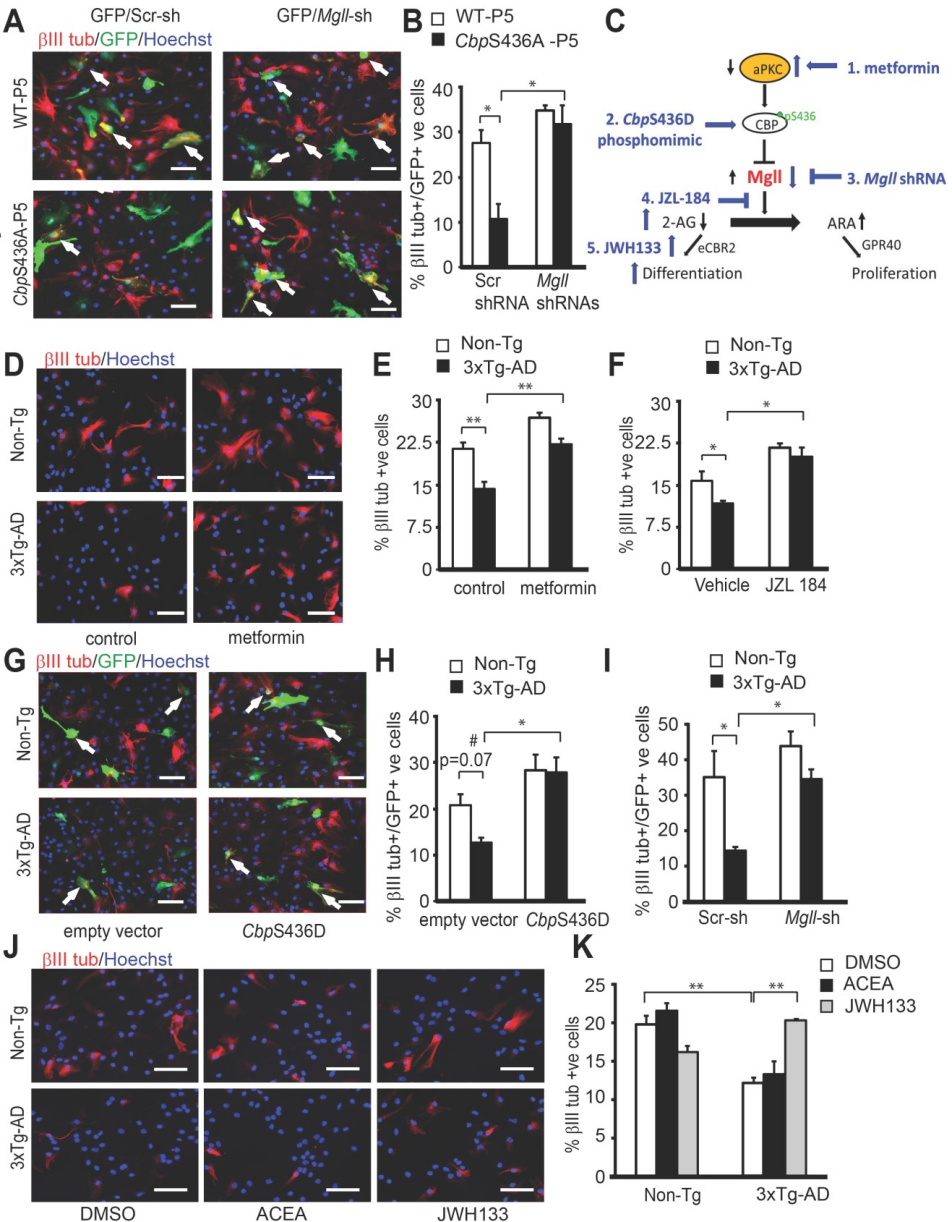
**Metformin rescues impaired neuronal differentiation of 3xTg-AD NPCs by modulating Mgll-regulated 2-AG-eCBR2 signaling. (A-B)** Representative immunofluorescent images (A) and quantification (B) of the percentage of βIII tubulin+/GFP+ neurons generated from differentiating P5 WT and *Cbp*S436A NPCs following transfection with Mgll or Scr shRNAs together with an eGFP plasmid, immunostained for GFP (green), βIII tubulin (red) and Hoechst (blue). Scale bar: 50 μm. Statistical analysis was performed using two-way ANOVA, n = 3 animals/group, * P < 0.05. **(C)** Schematic model depicting targeting of the aPKC-CBP mediated Mgll repression at multiple molecular levels to rescue the impaired pathway in 3xTg-AD NPCs. **(D)** Representative immunofluorescent images of differentiating P2 Non-Tg and 3xTg-AD NPCs, treated with control (water) or metformin and immunostained for βIII tubulin (red) and Hoechst (blue). Scale bar: 50 μm. **(E)** Quantification of the percentage of βIII tubulin+ neurons generated from P2 Non-Tg and 3xTg-AD NPCs 7 days upon differentiation in the absence and presence of metformin (500 nM). Data analysis was performed using two-way ANOVA, n = 3 animals/group, ** P < 0.01. **(F)** Quantification of the percentage of βIII tubulin+ neurons generated from P2 Non-Tg and 3xTg-AD NPCs 7 days upon differentiation in the presence of vehicle (0.1% DMSO) or JZL 184 (1 μM). Data analysis was performed using two-way ANOVA, n = 3 animals/group, * P < 0.05. **(G)** Representative immunofluorescent images of differentiating Non-Tg and 3xTg-AD NPCs, transfected with *Cbp*S436D or empty vector (pcDNA3.1) together with an eGFP plasmid, and immunostained for GFP (green), βIII tubulin (red) and Hoechst (blue). Scale bar: 50 μm. **(H)** Quantification of the percentage of βIII tubulin+/GFP+ neurons generated following transfection with *Cbp*S436D or pcDNA3.1 plasmid. Data were analysed by two-way ANOVA, n = 3 animals/group, * P < 0.05. **(I)** Quantification of the percentage of βIII tubulin+/GFP+ neurons generated from total transfected NPCs following transfection with Mgll or Scr shRNAs. Data were analysed by two-way ANOVA, n = 3 animals/group. * P < 0.05. **(J-K)** Representative immunofluorescent images (J) and quantitative analysis (K) of the percentage of βIII tubulin+ neurons generated from P2 Non-Tg and 3xTg-AD NPCs treated with eCBR1 and eCBR2 agonists, ACEA (1 µM) and JWH133 (1 µM), and vehicle (0.1% DMSO). Scale bar: 50 µm. Statistical analysis was performed using two-way ANOVA, n = 3 animals/group, ** P < 0.01. Data are represented as mean ± SEM. See also Figure S6.

Associated with this increased proliferation in P5 *Cbp*S436A NPCs and P2 3xTg-AD NPCs, we observed reduced neuronal differentiation of the P5 *Cbp*S436A NPCs and P2 3xTg-AD NPCs (Figure 6H-K, Student's t-test, * P < 0.05, ** P < 0.01). Since the P5 *Cbp*S436A NPCs display reduced neuronal differentiation (Figure 6H-I) concurrently with increased Mgll levels, we asked whether Mgll knockdown could rescue this differentiation deficit. To test this, we transfected P5 WT and *Cbp*S436A NPCs with *Mgll* or Scr shRNAs, together with an eGFP plasmid. As expected, we observed a significant differentiation deficit in the P5 *Cbp*S436A NPCs with Scr shRNA (control) transfection, which was rescued by knockdown of Mgll using *Mgll* shRNAs (Figure 7A-B, two-way ANOVA: Genotype x treatment F (1, 8) = 4.952, P = 0.0520; Genotype F (1, 8) = 8.556, P = 0.0191; Treatment F (1, 8) = 17.03, P = 0.0033, n = 12, Tukey's post-hoc test, * P < 0.05). These results confirm that elevated Mgll levels in the P5 *Cbp*S436A NPCs is responsible for their neuronal differentiation deficits.

### Metformin rescues impaired neuronal differentiation of 3xTg-AD NPCs by modulating Mgll-regulated 2-AG-eCBR2 signaling

To further assess if the impaired aPKC-CBP signaling is responsible for neuronal differentiation deficits observed in the 3xTg-AD NPCs, we examined whether reactivation of the pathway at multiple molecular levels could recover the differentiation defects of these 3xTg-AD NPCs (Figure 7C). First, we treated the differentiating P2 Non-Tg and 3xTg-AD NPCs with metformin (500 nM) to directly activate the aPKC-*Cbp* pathway and observed that metformin treatment rescued the 3xTg-AD NPC neuronal differentiation deficit, shown by a significant increase in the percentage of βIII tubulin+ neurons back to the basal levels observed in the Non-Tg NPCs (Figure 7D-E, two-way ANOVA: Genotype x treatment F (1, 8) = 1.532, P = 0.244; Genotype F (1, 8) = 34.29, P = 0.0002; Treatment F (1, 8) = 25.97, P = 0.0005, n = 12, Tukey's post-hoc test, ** P < 0.01). Since metformin is an AMP-dependent kinase (AMPK) activator that stimulates the aPKC-CBP pathway 24, we further confirmed that both 3xTg-AD and Non-Tg NPCs exhibit similar levels of AMPK protein expression in culture (Figure S6). Next, we transfected P2 Non-Tg and 3xTg-AD NPCs with the phosphomimic *Cbp*S436D plasmid to achieve a constitutive activation of the aPKC-CBP pathway before directing these NPCs towards differentiation. Overexpression of the phosphomimic *Cbp*S436D plasmid also significantly increased the percentage of βIII tubulin+ neurons from 3xTg-AD NPCs, rescuing their neuronal differentiation deficits (Figure 7G-H, two-way ANOVA: Genotype x treatment F (1, 8) = 1.674, P = 0.2318; Genotype F (1, 8) = 0.9419, P = 0.3602; Treatment F (1, 8) = 18.60, P = 0.0026, n = 12, Tukey's post-hoc test, * P < 0.05). Finally, we blocked Mgll activity and expression using a Mgll inhibitor (JZL 184, 1 µM) (Figure 7F, two-way ANOVA: Genotype x treatment F (1, 8) = 5.896, P = 0.0721; Genotype F (1, 8) = 10.88, P = 0.03; Treatment F (1, 8) = 13.69, P = 0.0208, n = 12, Tukey's post-hoc test, * P < 0.05) and *Mgll* shRNAs (Figure 7I, two-way ANOVA: Genotype x treatment F (1, 8) = 1.791, P = 0.2176; Genotype F (1, 8) = 12.64, P = 0.0074; Treatment F (1, 8) = 12.11, P = 0.0083, n = 12, Tukey's post-hoc test, * P < 0.05) respectively, in P2 3xTg-AD NPCs and observed a similar rescuing effect. Together, these data indicate that both reactivation of the aPKC-CBP pathway and removal of Mgll level/activity can alleviate the neuronal differentiation deficits in 3xTg-AD NPCs.

Since Mgll levels were upregulated in 3xTg-AD NPCs, we further asked whether 2-AG-eCBR signaling was involved in regulating 3xTg-AD neuronal differentiation defects. To test this, we treated P2 3xTg-AD NPCs with eCBR1 and eCBR2 agonists, ACEA (1 μM) and JWH133 (1 μM), respectively. Consistent with the previous results, we observed a significant reduction in the percentage of βIII tubulin+ neurons generated from P2 3xTg-AD NPCs in the vehicle (0.1% DMSO) group as compared to P2 Non-Tg NPCs treated with vehicle (Figure 7J-K). These differentiation defects were rescued upon treatment with eCBR2 agonist (JWH133) but not with eCBR1 agonist (ACEA) (Figure 7J-K, two-way ANOVA: Genotype x treatment F (2, 12) = 14.77, P = 0.0008; Genotype F (1, 12) = 14.12, P = 0.0032; Treatment F (2, 12) = 1.950, P = 0.1884, n = 18, Tukey's post-hoc test, ** P < 0.01). Thus, these data support that impairment of Mgll-regulated 2-AG-eCBR2 signaling is responsible for adult SVZ NPC neuronal differentiation deficit in the context of 3xTg-AD.

**Figure 8:**
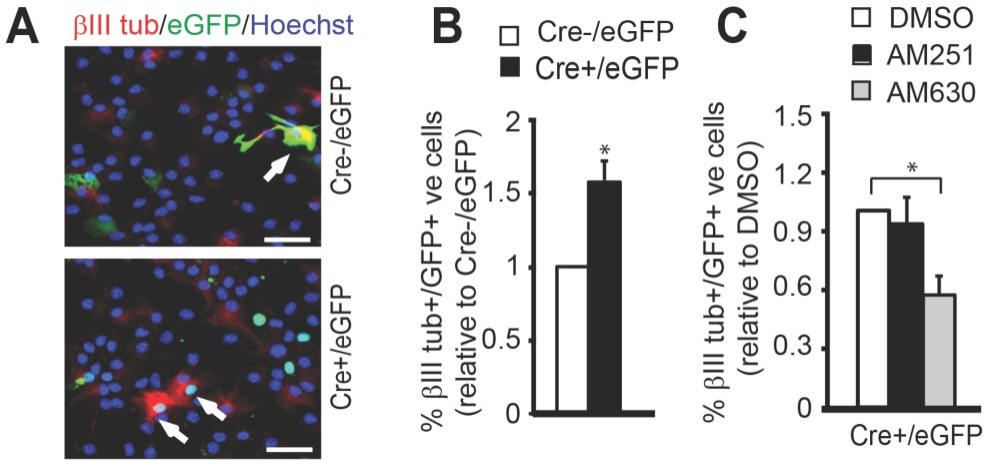
**Mgll-regulated 2-AG-eCBR2 signaling promote neuronal differentiation of adult SVZ NPCs. (A-B)** Representative immunofluorescent images (A) and quantification (B) of the percentage of βIII tubulin+/GFP+ neurons generated from Mgll-flxed NPCs following transfection with Cre+/eGFP or Cre-/eGFP plasmids 7 days upon differentiation. * P < 0.05. **(C)** Quantification of the percentage of βIII tubulin+/GFP+ neurons generated from Mgll-flxed NPCs following transfection with Cre+/eGFP (C) plasmids 7 days upon differentiation, treated with eCBR1 (AM251, 1 μM) and eCBR2 antagonists (AM630,1 μM), and vehicle (0.1% DMSO). Data were analysed by one-way ANOVA, n = 3 animals/group, * P < 0.05. Data are represented as mean ± SEM.

To further assess the role of 2-AG-eCBR signaling in mediating Mgll-regulated NPC neuronal differentiation under physiological conditions, we used *Mgll*-flxed mice to culture SVZ NPCs. To remove *Mgll* from these NPCs, we cultured and transfected *Mgll*-flxed NPCs with a Cre-eGFP (Cre+/eGFP) fusion plasmid and a Cre-negative eGFP (Cre-/eGFP) plasmid. We showed that Cre+/eGFP-transfected NPCs produced a significantly higher percentage of βIII tubulin+ neurons as compared to the Cre-/eGFP-transfected NPCs (Figure 8A-B, Student t-test, * P < 0.05). Interestingly, this increased neuronal differentiation in the Cre+/eGFP-transfected NPCs was eliminated in the presence of eCBR2 antagonist, AM630 (1 μM), but not eCBR1 antagonist, AM251 (1 μM) (Figure 8C, one-way ANOVA (F (2, 6) = 4.699, P = 0.059, n = 9, Dunnett's post-hoc test, * P < 0.05). Altogether, these data argue that Mgll-regulated 2-AG-eCBR2 signaling is essential to regulate adult SVZ NPC neuronal differentiation.

## Discussion

The present study demonstrates that aPKC-CBP mediated Mgll repression is important for appropriate NPC differentiation during healthy aging, and that when perturbed in AD, it causes impaired NPC function to generate fewer neurons, contributing to AD-associated memory decline and neuropathology. Specifically, these findings support five major conclusions. First, we show an aging-dependent accumulation of Mgll in both 3xTg-AD mouse model and human AD patient post-mortem hippocampal tissues. Second, we discover that elevated Mgll expression is caused by the attenuation of the aPKC-CBP pathway. This rapid accumulation of Mgll levels in the aging 3xTg-AD mice reduces the genesis of newborn neurons and perturbs spatial memory formation. Third, we show that metformin, an FDA-approved drug, reduces Mgll expression in aging 3xTg-AD mice to rescue reduced neuronal differentiation and prevent spatial memory decline by reactivating the aPKC-CBP pathway. Fourth, we demonstrate that the attenuation of the aPKC-CBP pathway in phospho-null *Cbp*S436A and 3xTg-AD NPCs results in elevated Mgll expression, which in turn exhibit increased NPC proliferation at the expense of neuronal differentiation. Finally, we find that metformin-induced aPKC-CBP pathway rescues neuronal differentiation deficits of 3xTg-AD NPCs by modulating Mgll regulated 2-AG-eCBR2 signaling.

Mgll is an important lipase that breaks down the endocannabinoid 2-AG to produce ARA, a major precursor for the inflammatory eicosanoids 46-48. Given its involvement in the regulation of these critical lipid signaling pathways with diverse physiological functions, Mgll has been considered a therapeutic target for neuroinflammatory and neurodegenerative disorders 9,49,50. Importantly, perturbed endocannabinoid signaling system is known to be involved in the acceleration of neuropathological and neurodegenerative conditions 51-53. For instance, early consumption of ethanol could dysregulate endocannabinoid signaling and accelerates cognitive impairment and β‐amyloid production in an APP/PSE animal model of AD 54. However, it remains to be determined how Mgll is regulated. In this regard, our study elucidates an aging-dependent epigenetic pathway that directly regulates Mgll expression at a transcriptional level. These findings thus pave the way for developing potential therapeutic approaches to repress Mgll expression by targeting the aPKC-CBP pathway.

Mgll activity shifts the balance between two critical bioactive lipid molecules: 2-AG and ARA. Interestingly, ARA-GPR40 signaling is known to promote NPC proliferation in the primate brain 45,55,56, while 2-AG-eCBR signaling has been shown to enhance neuronal differentiation of adult NPCs 57,58. In agreement with these earlier studies, our research identifies Mgll as a critical switch between NPC proliferation and differentiation by altering 2-AG-eCBR2 versus ARA-GPR40 signaling. This is intriguing as it indicates that two bioactive lipid signaling pathways that differentially regulate NPC proliferation and differentiation are controlled by a single lipase whose expression level can in turn be regulated by a signaling-directed epigenetic pathway, the aPKC-CBP pathway.

The aPKC-CBP pathway can be activated in response to various environmental stimuli. Our recent publication showed that this pathway is activated in the murine hippocampus when mice age from 3 to 6 months to sustain hippocampal neuronal differentiation and maturation and hippocampal-dependent memory 8. Intriguingly, the present work reveals that the aging-activated aPKC-CBP pathway is impaired in 3xTg-AD, an AD animal model, leading to reduced hippocampal neuronal differentiation/ maturation and perturbed spatial memory at the age of 8-10 months. Reduced aPKC activation has been extensively reported in AD patients 42-44, as well, Mgll activity has been associated with AD pathophysiology 10,11. However, a direct link between aPKC reduction and Mgll upregulation has never been considered in AD. In this regard, our study provides evidence to support a causal link between reduced aPKC activation and Mgll accumulation in AD, thereby providing mechanistic insight into the contribution of aPKC inactivation in AD predisposition. Importantly, our previous and current work argue that pathological aging impairs the aPKC-CBP pathway, leading to accumulated Mgll levels that perturb NPC differentiation and maturation early in life and potentially contribute to AD predisposition and memory decline.

Intriguingly, reactivation of the aPKC-CBP mediated Mgll repression at three molecular levels: aPKC, *Cbp*S436 and Mgll, all mitigate the neuronal differentiation deficits of 3xTg-AD NPCs to the same extent in culture. This strongly argues that targeting the aPKC-CBP mediated Mgll repression in AD has the capability to rescue impaired neurogenesis and improve memory. This was further proved in the current study through the use of the anti-diabetic drug metformin, a known AMPK activator. Notably, AMPK expression remains unaltered in 3xTg-AD NPCs, arguing that metformin is able to stimulate AMPK activity to reactivate aPKC-CBP mediated Mgll repression. Despite its hydrophilic nature, metformin can effectively cross the blood-brain barrier (BBB) and accumulate in various brain regions 59. As an organic cation, metformin has been demonstrated to be a super substrate for the organic cation transporter 3 (OCT3) which is expressed at the human BBB 60. Given its ability to actively pass through the BBB, metformin has become a promising reagent to treat CNS-based neurodegenerative disease. In particular, metformin has been well-studied for its potential role in treating AD. Recent studies show that metformin can reduce human Acetylcholinesterase (AChE) activity 61,62 which is well recognized as a therapeutic target against AD 62. However, the effects of metformin on neurodegenerative disease-related cognitive decline remain controversial 26,27 and therefore, a delineation of the molecular pathway through which metformin acts is imperative. Supported by our human AD post-mortem data and 3xTg-AD animal work, Mgll could be used as an early biomarker to identify AD patients for effective and early treatment with metformin. Our study provides the rationale to perform retrospective clinical research to identify a correlation between Mgll expression in peripheral blood and memory decline symptoms during AD progression in the absence and presence of metformin treatment. This retrospective study will then provide important information to properly design a prospective clinical trial where Mgll would serve as a biomarker in early stages of AD to identify potential metformin-responsive AD patients to restore their neurogenesis and spatial memory. This will bring personalized medicine to clinical practice.

In the present study, we also show that it is eCBR2 signaling, but not eCBR1 signaling, that mediates Mgll-regulated neuronal differentiation. These findings are intriguing given that a central characteristic of eCBR2 signaling is its involvement in neuroinflammatory responses in the central nervous system (CNS) 63,64, and that eCBR2 expression is often upregulated under neuropathological conditions such as AD 65,66. While a previous study showed that eCBR2 signaling is involved in neural progenitor proliferation 67, our findings tie eCBR2 signaling to Mgll-regulated neuronal differentiation of NPCs, highlighting the important role of eCBR2 signaling in regenerating newborn neurons in the context of neurodegenerative disease.

Finally, while this study effectively demonstrates Mgll's role in aging-dependent AD pathophysiology and its potential use as a biomarker for effective metformin treatment, yet there are certain limitations. While our results show altered Mgll expression in AD hippocampal tissues, associated lipidomic changes in relation to this altered Mgll expression have not been assessed. Characterization of ultimate lipid metabolism outcomes in response to changes in Mgll levels will require additional studies. Furthermore, an examination of the effect of metformin in an animal AD model where Mgll is not elevated, is also required. We posit that this kind of study will address the specificity of Mgll as a biomarker to identify a subpopulation of AD patients who will respond to metformin to prevent memory decline and restore neurogenesis. In light of our study and metformin as an FDA-approved drug, we are well-positioned to initiate a clinical trial to examine *Mgll* mRNA levels in AD patients' peripheral blood as a screening methodology to identify patients to receive effective metformin treatment.

## Supplementary Material

Supplementary figures and tables.

## Abbreviations

2-AG: 2-arachidonoyl glycerol
ACEA: Arachidonyl-2'-chloroethylamide
AChE: acetylcholinesterase
AD: Alzheimer's disease
3xTg-AD: triple transgenic model of Alzheimer's disease
AMPK: AMP-dependent kinase
ANOVA: analysis of variance
aPKC: atypical protein kinase C
ARA: arachidonic acid
Aβ: β-amyloid
BBB: blood-brain barrier
BrdU: 5-Bromo-2´-deoxyuridine
BSA: bovine serum albumin
CBP: CREB-binding protein
ChIP: chromatin immunoprecipitation
CM: conditioned medium
CNS: central nervous system
Co-IP: co-immunoprecipitation
DAB: 3,3-Diaminobenzidine
DCX: doublecortin
DG: dentate gyrus
DIV: days *in vitro*
DMSO: dimethyl sulfoxide
eCBR1: endocannabinoid receptor 1
eCBR2: endocannabinoid receptor 2
EdU: 5-Ethynyl-2'-deoxyuridine
eGFP: enhanced green fluorescent protein
GAPDH: Glyceraldehyde 3-phosphate dehydrogenase
GPR40: G-protein-coupled receptor 40
HAT: histone acetyltransferase
i.p.: intraperitoneal
IHC: immunohistochemistry
Mgll: monoacylglycerol lipase
MWM: Morris water maze
NeuN: neuronal nuclei
NPC: neural stem/progenitor cell
NS: neurosphere
OCT3: organic cation transporter 3
P2: passage 2
P5: passage 5
p-aPKC: phosphorylated atypical protein kinase C
SGZ: subgranular zone
shRNA: short-hairpin RNA
SVZ: subventricular zone
T-aPKC: total atypical protein kinase C
WT: wildtype
