# Supplementary material for: Dysregulated expression of monoacylglycerol lipase is a marker for anti-diabetic drug metformin-targeted therapy to correct impaired neurogenesis and spatial memory in Alzheimer's disease
Source: Theranostics. 2020 May 15;10(14):6337–60. doi: 10.7150/thno.44962 (PMC7255032; doi:10.7150/thno.44962)
Supplement: Supplementary file 1 — Supplementary figures and tables. [file thnov10p6337s1.pdf]

SUPPLEMENTAL ITEMS:

FigureS1. **Mgll protein levels in 3xTg-AD and *Cbp*S436A mice hippocampal DG and human post-mortem AD hippocampal DG tissues.**

Figure S2. **JZL 184 and** m**etformin do not significantly change general motor activities of WT and *Cbp*S436A, and Non-Tg and 3xTg-AD mice.**

Figure S3. **Metformin treatment removes intracellular β-amyloid accumulation from 3xTg hippocampal dentate gyrus.**

Figure S4. **(A) ChIP-qPCR analysis for CBP binding at Mgll promoter in differentiating WT and *Cbp*S436A NPCs in the presence of metformin;** (**B-D) Efficiency of shRNA-mediated Mgll knockdown.**

Figure S5. **Continued passaging does not change total aPKC protein levels.**

Figure S6. **AMPK protein expression in 3xTg-AD and Non-Tg NPCs.**

Table S1. **Human post-mortem samples information regarding age, sex and post-mortem delay.**

Table S2. **RNA-seq analysis of differentiating WT and *Cbp*S436A NPCs in the presence of metformin.**

Supplementary Materials:

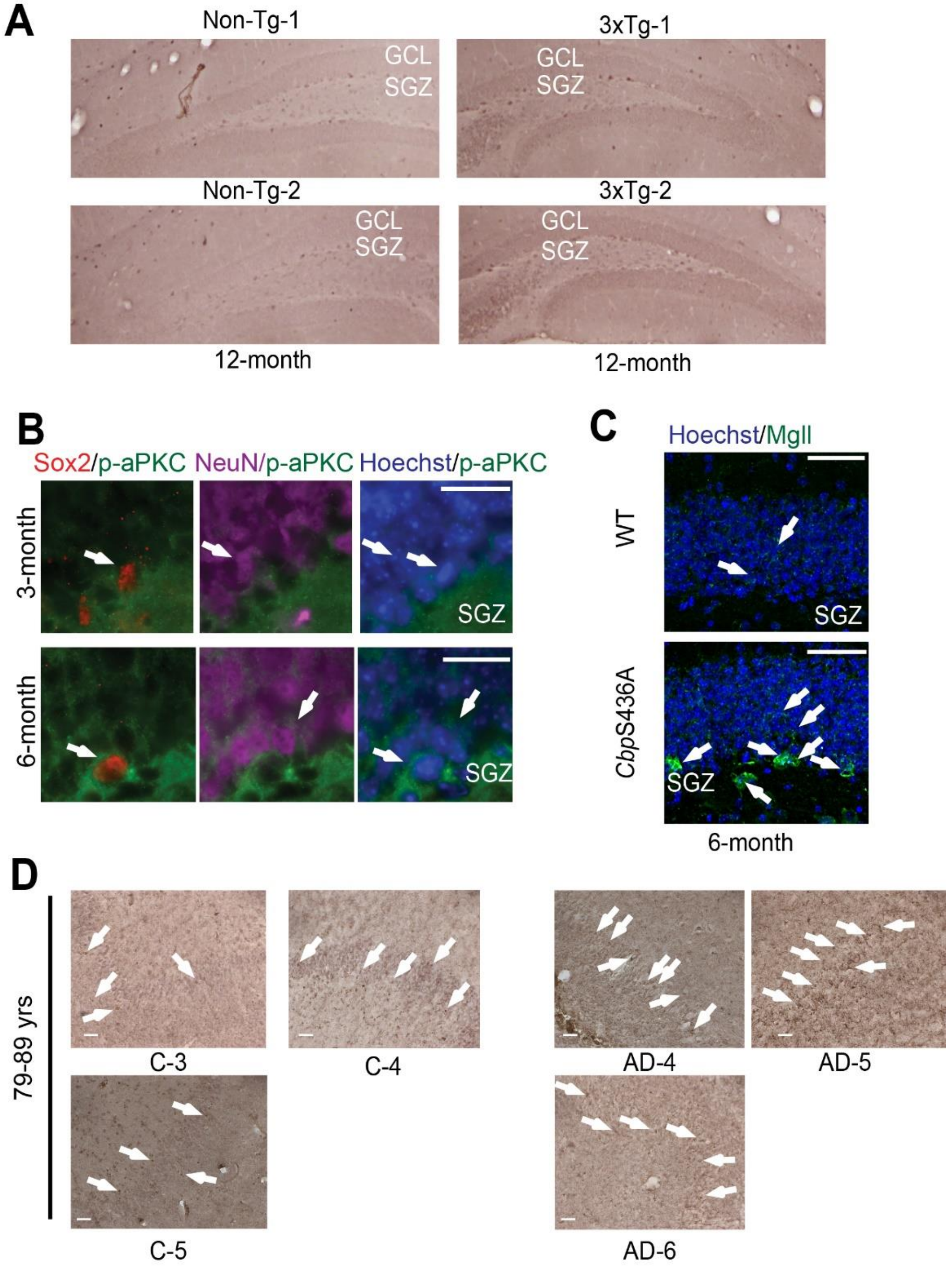

Figure S1. Mgll protein levels in 3xTg-AD and *Cbp*S436A mice hippocampal DG and human post-mortem AD hippocampal DG tissues. (A) Images of 12-month-old Non-Tg and 3xTg-AD hippocampal DG sections immunostained for Mgll with DAB staining (n=2 animals/genotype). (B) Images of hippocampal DG (SGZ/GCL layers) sections from 3-month-old and 6-month-old WT mice, immunostained for Sox2 (red), NeuN (purple) and p-aPKC (green) and counterstained with Hoechst (blue). Arrows denote NeuN+ or Sox2+ cells; scale bar: 25 µm. (C) Images of hippocampal DG sections from 6-month-old WT and *Cbp*S436A mice, immunostained for Mgll (green) and counterstained with Hoechst (blue). Arrows denote Mgll+ cells; scale bar: 25 µm. (D) Images of human hippocampal DG sections from AD patients and their age-matched healthy controls (79-89 years) following Mgll immunohistochemistry with DAB staining. Arrows denote Mgll+ DAB stained cells. Scale bar: 50 µm.

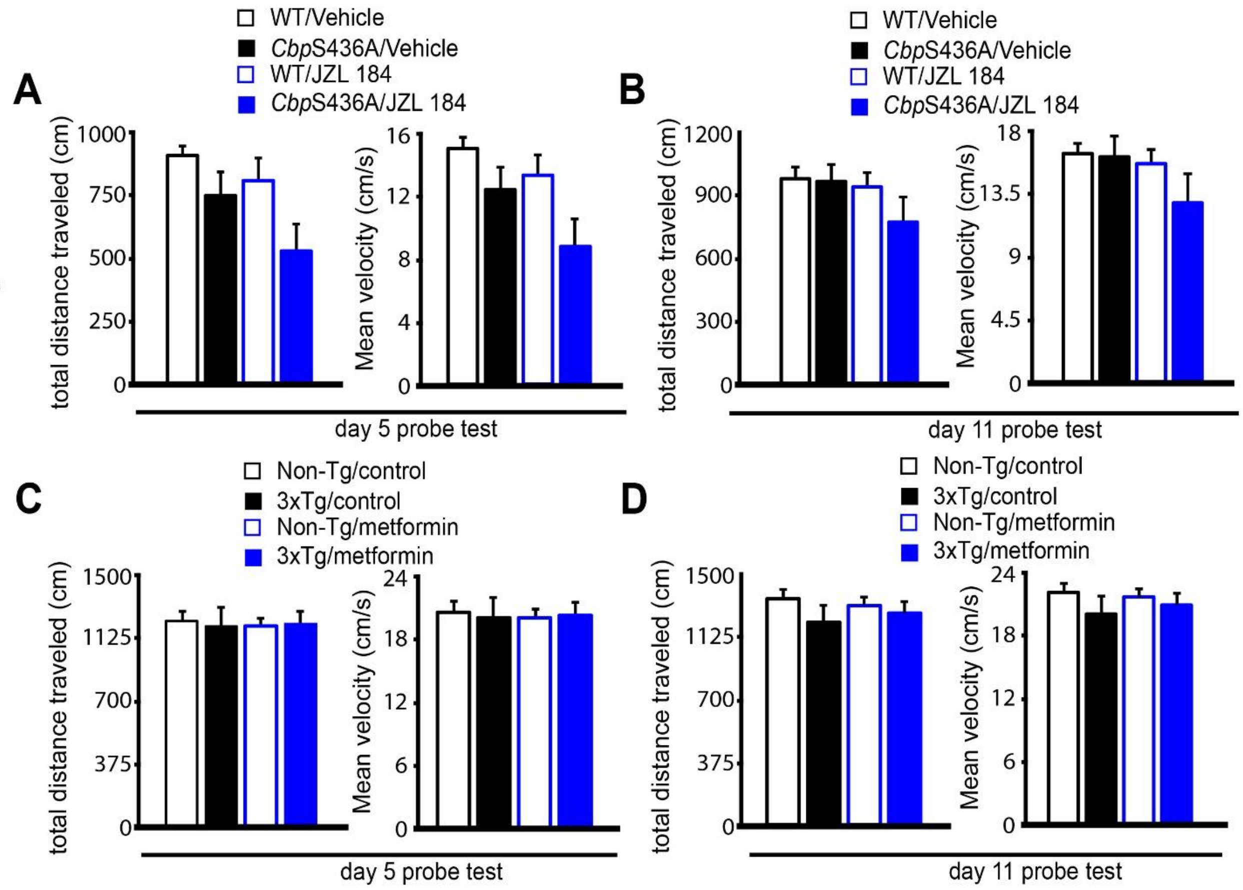

Figure S2. JZL 184 and metformin does not significantly change general motor activities of WT and *Cbp*S436A, and Non-Tg and 3xTg-AD mice, respectively. (A-B) Analysis of the total distance travelled and the mean velocity during short-term MWM probe test (A, day 5) and long-term MWM probe test (B, day 11) for 6-month WT and *Cbp*S436A mice that received either vehicle or JZL 184 treatment. (C-D) Analysis of the total distance travelled and the mean velocity during short-term MWM probe test (C, day 5) and long-term MWM probe test (D, day 11) for Non-Tg and 3xTg-AD mice that received either control or metformin treatment.

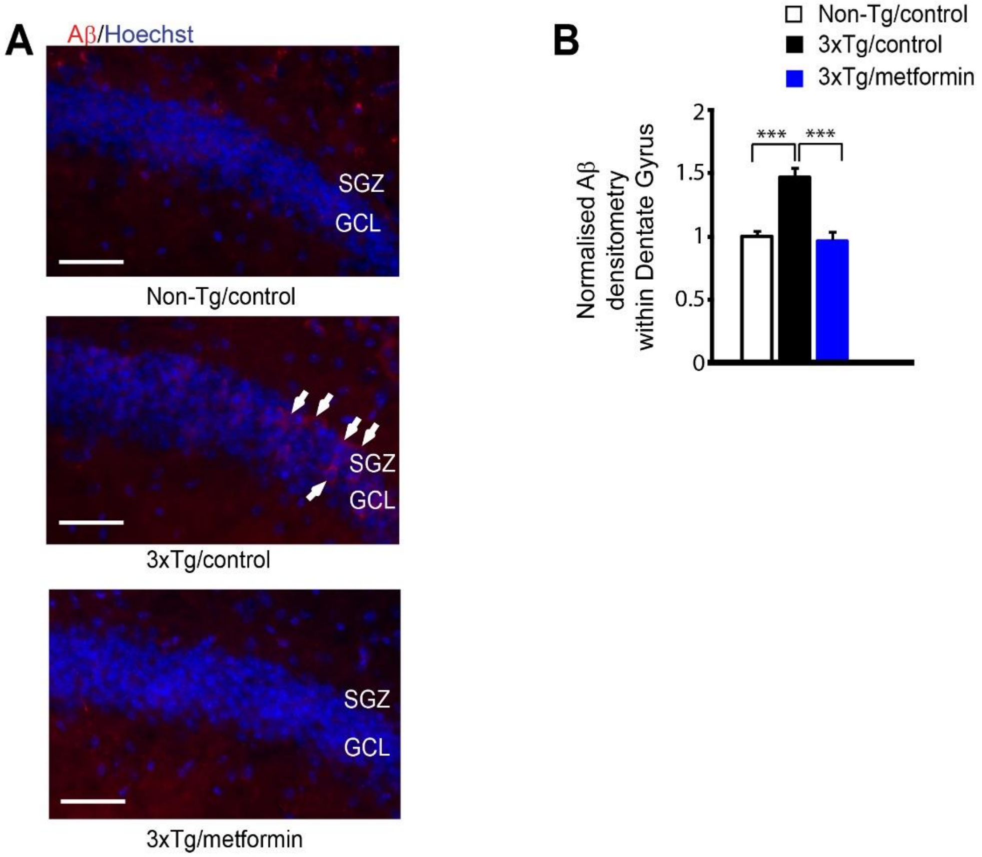

Figure S3. Metformin treatment removes intracellular β-amyloid accumulation from 3xTg hippocampal dentate gyrus. (A-B) Quantification by densitometry of β-amyloid (Aβ) immunoreactivity in the hippocampal dentate gyrus as in **A**; measurements were normalized to one of sections from Non-Tg/control group. (n=20-30 section/group). Data analysis was performed using one-way ANOVA ($F(2,136) = 34.42$, $P < 0.0001$, n=139) *** $p < 0.001$. Scale bar: 100 µm.

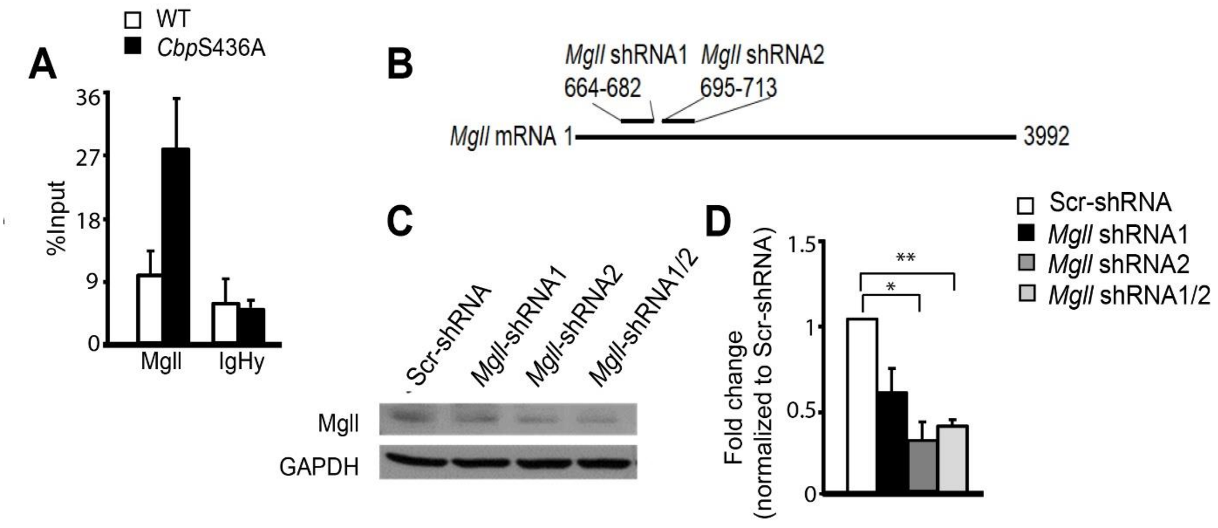

Figure S4. (A) **ChIP-qPCR analysis for CBP binding at Mgll promoter in differentiating WT and *Cbp*S436A NPCs in the presence of metformin.** ChIP analysis was performed to analyze the enrichment of CBP at the *Mgll* promoter. IgHy enhancer was used as a negative control. (n=3 animals/group). **(B-D) Efficiency of shRNA-mediated Mgll knockdown.** (B) Schematic representing Mgll shRNAs target regions. (C) Representative western blot images of total protein lysates from NIH3T3 cells transfected with *Mgll* shRNAs and a Scr shRNA, probed for Mgll and GAPDH (a loading control). (D) Quantitative analysis of Mgll expression in NIH3T3 cells 48 h after shRNA transfection, normalized to GAPDH. Data from 3 independent experiments was analyzed using one-way ANOVA ($F(3, 8) = 10.09$, $P = 0.0043$) with Dunnett's post-hoc test. * $P < 0.05$, ** $P < 0.01$. (Scr sh: Scramble shRNA; Mgll sh1: Mgll shRNA 1; Mgll sh2: Mgll shRNA 2).

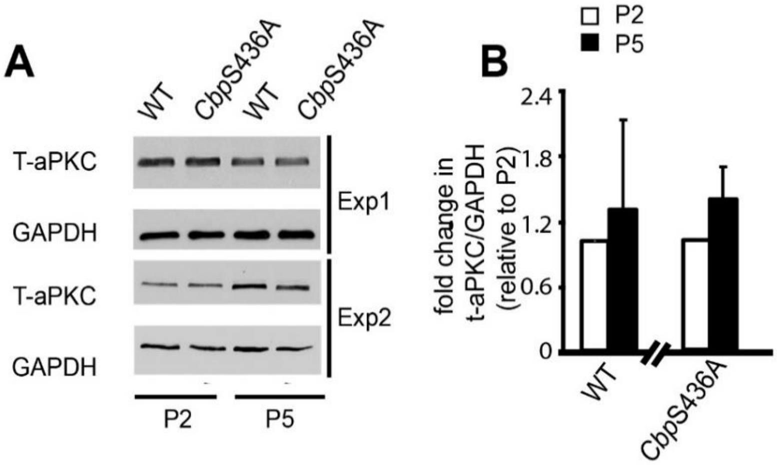

Figure S5. Continued passaging does not change total aPKC protein levels. (A) Representative western blot images of total protein lysates from proliferating P2 (early passage) and P5 (late passage) WT and *Cbp*S436A NPCs, probed for total aPKC and GAPDH (a loading control) from experiment1 (top panel) and 2 (bottom panel). (B) Quantitative analysis of total aPKC expression, relative to GAPDH in proliferating P2 and P5 WT and *Cbp*S436A NPCs. Data was normalised to corresponding P2 NPCs for each genotype.

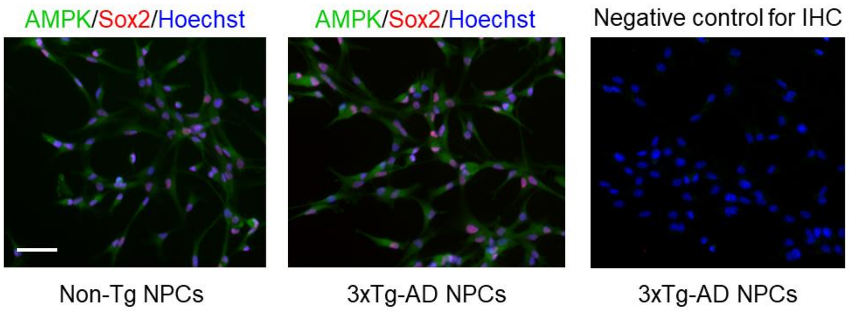

Figure S6. AMPK protein expression in 3xTg-AD and Non-Tg NPCs. Immunofluorescent images of cultured P2 NPCs from 3xTg-AD and Non-Tg mice, immunostained for Sox2 (red) and AMPK (green), counterstained for Hoechst (blue). Negative control for IHC image was from 3xTg-AD NPCs that received only secondary antibody incubation. Scale bar: 50 µm.

		Age	sex	post-mortem (hour)
Healthy control	C-1	61	M	12
	C-2	75	M	24
	C-3	81	F	18
	C-4	80	F	18
	C-5	89	M	20
AD patients	AD-1	59	M	4
	AD-2	70	M	26
	AD-3	76	M	24
	AD-4	79	F	9
	AD-5	81	F	6
	AD-6	84	M	13

Table S1. Human post-mortem samples information regarding age, sex and post-mortem delay in tissue fixation. Human DG sections were stored in antifreeze solution (40% PBS, 30% Glycerol) at -20°C.

gene_short_name	log2_fold_change	Ensembl ID	Species	Gene Name
9930013L23Rik	1.27506	ENSMUSG00000052353	Mus musculus	RIKEN cDNA 9930013L23 gene
A2m	0.807783	ENSMUSG00000030111	Mus musculus	alpha-2-macroglobulin
Abca9	1.18481	ENSMUSG00000041797	Mus musculus	ATP-binding cassette, sub-family A (ABC1), member 9
Atp1a2	1.16923	ENSMUSG00000007097	Mus musculus	ATPase, Na+/K+ transporting, alpha 2 polypeptide
Cdkn1c	2.20316	ENSMUSG00000037664	Mus musculus	cyclin-dependent kinase inhibitor 1C (P57)
Chgb	2.04785	ENSMUSG00000027350	Mus musculus	chromogranin B
Chl1	1.35974	ENSMUSG00000030077	Mus musculus	cell adhesion molecule with homology to L1CAM
Col18a1	2.08565	ENSMUSG00000001435	Mus musculus	collagen, type XVIII, alpha 1
Csrnp1	-1.21314	ENSMUSG00000032515	Mus musculus	cysteine-serine-rich nuclear protein 1
Dio2	1.66036	ENSMUSG00000007682	Mus musculus	deiodinase, iodothyronine, type II
Dmrta2	3.00771	ENSMUSG00000047143	Mus musculus	doublesex and mab-3 related transcription factor like family A2
Dusp5	-1.1296	ENSMUSG00000034765	Mus musculus	dual specificity phosphatase 5
Egr1	-1.32754	ENSMUSG00000038418	Mus musculus	early growth response 1
Egr2	-1.93359	ENSMUSG00000037868	Mus musculus	early growth response 2
Eps8l1	4.1267	ENSMUSG00000006154	Mus musculus	EPS8-like 1
Fam178a	0.864121	ENSMUSG00000036097	Mus musculus	family with sequence similarity 178, member A
Fbln5	1.43958	ENSMUSG00000021186	Mus musculus	fibulin 5
Fosb	-2.72036	ENSMUSG00000003545	Mus musculus	FBJ osteosarcoma oncogene B
Gad1	3.09252	ENSMUSG00000070880	Mus musculus	glutamic acid decarboxylase 1
Gfap	0.820592	ENSMUSG00000020932	Mus musculus	glial fibrillary acidic protein
Gria1	0.754434	ENSMUSG00000020524	Mus musculus	glutamate receptor, ionotropic, AMPA1 (alpha 1); similar to Glutamate receptor, ionotropic, AMPA1 (alpha 1)
Gucy1a3	1.69036	ENSMUSG00000033910	Mus musculus	guanylate cyclase 1, soluble, alpha 3
Hopx	1.61918	ENSMUSG00000059325	Mus musculus	HOP homeobox
Lmx1a	4.10731	ENSMUSG00000026686	Mus musculus	LIM homeobox transcription factor 1 alpha
Maf	0.860591	ENSMUSG00000055435	Mus musculus	similar to c-Maf long form; avian musculoaponeurotic fibrosarcoma (v-maf) AS42 oncogene homolog
Mgll	1.51722	ENSMUSG00000033174	Mus musculus	monoglyceride lipase
Mki67	1.12498	ENSMUSG00000031004	Mus musculus	antigen identified by monoclonal antibody Ki 67
Nkain2	-1.14397	ENSMUSG00000069670	Mus musculus	Na+/K+ transporting ATPase interacting 2
Nr4a1	-1.59272	ENSMUSG00000023034	Mus musculus	nuclear receptor subfamily 4, group A, member 1
Pbk	1.73027	ENSMUSG00000022033	Mus musculus	PDZ binding kinase
Pcp4l1	0.948439	ENSMUSG00000038370	Mus musculus	Purkinje cell protein 4-like 1
Per1	-0.754279	ENSMUSG00000020893	Mus musculus	period homolog 1 (Drosophila)
Pgm5	1.99293	ENSMUSG00000041731	Mus musculus	phosphoglucomutase 5
Pmaip1	-1.53678	ENSMUSG00000024521	Mus musculus	phorbol-12-myristate-13-acetate-induced protein 1
Prc1	1.10732	ENSMUSG00000038943	Mus musculus	protein regulator of cytokinesis 1
Rbp1	-1.23272	ENSMUSG00000046402	Mus musculus	retinol binding protein 1, cellular
Sh3bp5	1.04966	ENSMUSG00000021892	Mus musculus	SH3-domain binding protein 5 (BTK-associated)
Slc6a15	-1.02873	ENSMUSG00000019894	Mus musculus	solute carrier family 6 (neurotransmitter transporter), member 15
Sytl2	0.87338	ENSMUSG00000030616	Mus musculus	synaptotagmin-like 2
Tagln3	0.91303	ENSMUSG00000022658	Mus musculus	transgelin 3
Thy1	1.02941	ENSMUSG00000032011	Mus musculus	thymus cell antigen 1, theta
Tmem163	1.802	ENSMUSG00000026347	Mus musculus	hypothetical protein LOC100047091; transmembrane protein 163
Top2a	1.17366	ENSMUSG00000020914	Mus musculus	topoisomerase (DNA) II alpha
Wbscr17	1.44404	ENSMUSG00000034040	Mus musculus	Williams-Beuren syndrome chromosome region 17 homolog (human); similar to UDP-GalNAc:polypeptide N-acetylgalactosaminyltransferase-like 3
Wisp2	0.933059	ENSMUSG00000027656	Mus musculus	WNT1 inducible signaling pathway protein 2
Zic1	1.6321	ENSMUSG00000032368	Mus musculus	similar to Zic protein; zinc finger protein of the cerebellum 1
Zic2	1.65671	ENSMUSG00000061524	Mus musculus	zinc finger protein of the cerebellum 2
Zic3	1.94374	ENSMUSG00000067860	Mus musculus	zinc finger protein of the cerebellum 3

Table S2. RNA-seq analysis from differentiating WT and *Cbp*S436A NPCs in the presence of metformin for 6 days.
